# Sustainable Ellagitannin-Rich Pomegranate Peel Extracts Activate SKN-1/NRF2-Dependent Antioxidant Responses and Promote Healthy Aging in *Caenorhabditis elegans*

**DOI:** 10.3390/antiox15081018

**Published:** 2026-08-15

**Authors:** Emily Schifano, Francesco Cairone, Marco Guarnieri, Andrea Bonfanti, Patrizia Mancini, Irene Arpante, Arianna Montanari, Cristina Mazzoni, Daniela Uccelletti, Stefania Cesa

**Affiliations:** 1 Department for Well-Being, Health and Environmental Sustainability—BeSSA, Sapienza University of Rome, Via della Fontanelle, 02100 Rieti, Italy; emily.schifano@uniroma1.it; 2 Department of Chemistry and Technologies of Drug, Sapienza University of Rome, Piazzale Aldo Moro 5, 00185 Rome, Italy; irene.arpante@uniroma1.it (I.A.); stefania.cesa@uniroma1.it (S.C.); 3 Department of Biology and Biotechnologies, Sapienza University of Rome, Piazzale Aldo Moro 5, 00185 Rome, Italy; marco.guarnieri@uniroma1.it (M.G.); andrea.bonfanti@uniroma1.it (A.B.); ari.montanari@uniroma1.it (A.M.); cristina.mazzoni@uniroma1.it (C.M.); 4 Department of Experimental Medicine, Sapienza University of Rome, Viale Regina Elena 324, 00161 Rome, Italy; patrizia.mancini@uniroma1.it

**Keywords:** pomegranate peel, ellagitannins, *Caenorhabditis elegans*, oxidative stress, SKN-1/NRF2, antioxidant activity

## Abstract

The valorization of food by-products represents a key strategy for sustainable food systems and the circular bioeconomy. Pomegranate peels from two selected Italian cultivars were subjected to environmentally friendly extraction processes aimed at optimizing ellagitannins’ recovery and exploiting their biological activity. Punicalagin (PU) was the predominant compound, and the extracts exhibited high antioxidant capacity in both DPPH and FRAP assays. The Granato cultivar was selected for in vivo evaluation on the *Caenorhabditis elegans* (*C. elegans*) nematode model, where it extended lifespan and improved key healthspan markers. Locomotion, feeding activity, muscle integrity, and intestinal barrier function were improved through the activation of conserved stress-response pathways, particularly the SKN-1/NRF2 axis. The results were associated with improved mitochondrial function and reduced age-related oxidative stress, consistent with a hormetic mechanism. The extract also exhibited antibacterial activity, mainly against Gram-positive bacteria. Overall, the results highlight pomegranate peel as a sustainable source of high-value bioactive compounds with potential applications in nutrition, dietary supplements, and dermo-cosmetics.

## 1. Introduction

Growing environmental consciousness and the urgent need to reduce waste materials throughout the agri-food chain are orienting researchers towards production models inspired by the circular economy, in which food by-products are no longer considered waste but rather resources with high functional value [1]. In this context, pomegranate peel (*Punica granatum* L.), accounting for up to 50% of the fruit’s weight, is of great interest to the food and nutritional industry for its richness in bioactive polyphenols, namely ellagitannins, punicalagin (PU) and ellagic acid (EA) [2,3].

These compounds show strong antioxidant, anti-inflammatory, and antimicrobial properties, making pomegranate peel a promising by-product for the development of functional ingredients with preservative, protective, and health-promoting properties [4].

Peel extracts can effectively inhibit the growth of several Gram-positive and Gram-negative foodborne pathogens, pointing to their potential use as alternative antimicrobial agents to address the growing concern of antibiotic resistance [5,6].

However, the standardization of extraction processes remains a crucial challenge for the industrial transfer of these functional ingredients. Current research is focused on green strategies based on the use of food-safe solvents, short times and mild temperatures, and low energy impact, in line with the principles of sustainable chemistry and circular bioeconomy [7]. Solvent choice and operating conditions are crucial not only for extraction yields but also to preserve the quality of the contained bioactive compounds, namely ellagitannins, which are often susceptible to oxidative processes [8].

The role of ellagitannins in modulating biological processes associated with cellular aging and oxidative stress has been increasingly documented, due to their ability to neutralize reactive oxygen species (ROS) and interact with key molecular pathways related to longevity [9]. Among biological models, *Caenorhabditis elegans* (*C. elegans*) represents a powerful in vivo system for evaluating the cytoprotective and anti-aging potential of plant-derived bioactives [10]. This free-living nematode offers several advantages for nutraceutical and toxicological research, including a short and well-defined lifespan, reproducible physiological responses, and easy maintenance under controlled laboratory conditions [11]. Importantly, despite its simplicity, *C. elegans* shares conserved molecular pathways with humans involved in stress resistance, immunity, metabolism, and longevity, including insulin/IGF-1 (DAF-2/DAF-16), SKN-1/Nrf2, and HSF-1 signaling pathways [12].

These characteristics make it particularly suitable for elucidating the mechanisms through which polyphenol-rich extracts exert antioxidant or pro-longevity effects. Its transparency and the availability of fluorescent reporter strains allow direct evaluation of cellular responses to oxidative stress, including ROS accumulation, activation of detoxification enzymes, and mitochondrial alterations [13].

Survival assays, motility measurements, and fertility tests can also be rapidly performed, enabling detection of beneficial or adverse effects of bioactive compounds. Consequently, *C. elegans* has become a widely used model for screening natural compounds with potential health-promoting properties and elucidating their mechanisms of action in vivo. In parallel, increasing attention has been devoted to the use of natural polyphenols in dermo-cosmetic applications due to their ability to modulate oxidative stress, inflammatory responses, and skin-aging-related pathways, particularly through NRF2-mediated cytoprotective mechanisms and mitochondrial preservation under photooxidative stress conditions [14,15].

These properties make polyphenol-rich extracts attractive candidates for the development of antioxidant and anti-photoaging dermo-cosmetic formulations [16].

Based on this background, the present study focused on two Italian pomegranate cultivars, Roce and Granato, selected because previous studies identified them as Southern Italian genotypes with distinct pomological and phytochemical characteristics, with Granato showing one of the highest nutraceutical values among Italian accessions [17,18]. Since cultivar-dependent variability may influence both ellagitannin recovery and biological activity, different sustainable extraction strategies were compared to evaluate extraction yield, phenolic composition, antioxidant and antimicrobial activities, and the in vivo effects of the extracts in *C. elegans*.

The aim was to identify the most promising cultivar extraction strategy for the functional valorization of pomegranate peel and to elucidate the molecular mechanisms underlying its antioxidant and pro-longevity effects in vivo, thereby supporting the development of pomegranate-derived ingredients for nutritional and dermo-cosmetic applications.

## 2. Materials and Methods

### 2.1. Materials

The solvents (formic acid, 99%, acetic acid, 99%, acetonitrile, 99.9%, ethanol, 96%, and methanol, 99.8%), the analytical reagents 2,2-diphenyl-1-picrylhydrazyl (DPPH), the Ferric Reducing Antioxidant Power (FRAP) Assay Kit and the reference standards for gallic acid, ellagic acid and punicalagin were supplied by Merck Science Life srl. (Milan, Italy). Bidistilled water was provided by Carlo Erba (Milan, Italy). Pomegranate fruits belonging to two cultivars, Roce (R) and Granato (G), were kindly gifted by the organic farm Giovomel (Aiello del Sabato, Avellino, Italy).

### 2.2. Sample Preparation

The pomegranate fruits were kindly donated by the organic farm Giovomel (Aiello del Sabato, Avellino, Italy). They were harvested during the commercial ripening season (approximately October), corresponding to full fruit maturity. After harvesting, the fruits were washed, manually peeled, and the peels immediately stored at −20 °C until extraction. After thawing, the peels were homogenized using a domestic mixer (Girmi^®^ TR01, Bergamo, Italy), further homogenized using an Ultraturrax^®^ (Beckman J-21B, Milan, Italy) and subsequently subjected to different extraction procedures.

### 2.3. Aqueous, Ethanolic and Hydroalcoholic Extraction

Five grams of homogenized peels were extracted with distilled water or with 96% ethanol or with a hydroalcoholic mixture (EtOH:H_2_O 70:30 *v*/*v* at a 1:3 *w*/*v* ratio). Extraction was performed under continuous stirring at room temperature for 1 h, protecting from light. The mixture was then filtered under vacuum using a Büchner funnel. The aqueous extracts (RA, GA) were freeze-dried with a LIO5P, 4K, equipped with a BOC Edwards RV pump (Milan, Italy), and the ethanolic (RE, GE) and hydroalcoholic (RH, GH) extracts were evaporated to dryness. Freeze-dried or dried samples were stored at 4 °C until further analyses were performed.

### 2.4. HPLC-DAD Analysis

The previously obtained solid extracts were dissolved in hydroalcoholic mixtures at a concentration of 5 mg/mL and filtered using 0.45 µm syringe filters (25 mm diameter; LLG LABWARE, Meckenheim, Germany). HPLC-DAD (High Performance Liquid Chromatography coupled with Diode Array Detection) analyses were performed using a Perkin-Elmer system (Waltham, MA, USA) equipped with an LC Series 200 pump, a Series 200 DAD detector (LC200D), a Series 200 autosampler, and TotalChrom software v. 6.3.2 for data processing. The HPLC method was optimized based on the procedure reported by Lasalvia et al. (2022) [19]. The separation was achieved on an RP18 column (NUCLEODUR Sphinx^®^, Macherey-Nagel GmbH & Co, Düren, Germany, 5 µm), using a mobile phase consisting of water acidified with 5% formic acid (A) and acetonitrile (B) in gradient mode. The injection volume was 10 µL, and the flow rate was 1 mL/min. The applied gradient program was as follows: from 100% to 45% of A over 50 min, then from 45% to 20% A over 10 min. Chromatograms were recorded at 360 nm for the identification of ellagitannins. The HPLC-DAD method was validated in terms of linearity, limit of detection (LOD), limit of quantification (LOQ), specificity, and precision. Calibration curves obtained with external reference standards showed excellent linearity (R^2^ > 0.99). Method precision, evaluated through repeated injections at different concentration levels, resulted in %RSD values below 5% for both intra- and inter-day analyses. Matrix effects were minimized by sample filtration and by applying external calibration under the same chromatographic conditions. Specifically, quantification was performed using calibration curves of punicalin (PUN) (y = 8.6x − 12.7; R^2^ = 0.9974, in the range 10–1000 mg/L, LOD 5 mg/L, LOQ 20 mg/L), punicalagin (PU) (y = 5.1x − 56.5; R^2^ = 0.9998; range 0.06–1.14 mg/mL; LOD 0.0059 mg/mL; LOQ 0.018 mg/mL) and ellagic acid (EA) (y = 13.8x + 40.4; R^2^ = 0.9992; range 0.01–1.11 mg/mL; LOD 0.0018 mg/mL; LOQ 0.0055 mg/mL), with results expressed in mg/g of dried extract (DE).

### 2.5. DPPH (2,2-diphenyl-1-picrylhydrazyl) Assay

Antiradical activity of pomegranate peel extracts was tested using the 2,2-diphenyl-1-picrylhydrazyl (DPPH) assay. The DPPH reagent was dissolved at a concentration of 100 μM, stored at 4 °C, and protected from light, while monitoring the radical stability at 517 nm over time. The obtained freeze-dried or dried samples were dissolved in 96% ethanol to a final concentration of 1 mg/mL. An aliquot of 50 μL of each sample was added to 950 μL of the stock DPPH solution, and the degradation kinetics were monitored in real time at the same wavelength of 517 nm for 30 min, at room temperature. Spectrophotometric results were expressed as mg/g gallic acid equivalents, calculated using the calibration curve of gallic acid [y = −0.273 ln(x) − 1.1712, R^2^ = 0.9982], in the range 0.06–0.8 mg/mL.

### 2.6. FRAP (Ferric Ion Reducing Antioxidant Power) Assay

Antioxidant activity of pomegranate peel extracts was tested using the Ferric Reducing Antioxidant Power (FRAP) assay. The reagent solution was prepared using the FRAP Assay Kit (Merck Science Srl, Milan, Italy), composed of FRAP Assay Btheer, FRAP Probe, and FeCl_3_. A 10 mL FRAP solution was prepared at a 1:10 *v*/*v* ratio, and the reagent stability was confirmed at 539 nm over time. Pomegranate peel extracts were dissolved in distilled water to a final concentration of 1 mg/mL. An aliquot of 50 μL of each sample was added to 950 μL of the FRAP solution, and the reduction kinetics were monitored in real time at the same wavelength of 539 nm for 30 min, at room temperature. Spectrophotometric results were expressed as mg/g ellagic acid equivalents, calculated on the calibration curve of ellagic acid [y = 0.1093 ln(x) + 0.9291, R^2^ = 0.9955], in the range 0.46–23 µg/mL.

### 2.7. Cell Viability Assay on HaCaT Cells

Cell viability was evaluated using the MTT colorimetric assay. HaCaT cells were seeded into 96-well plates at a density of 3 × 10^3^ cells per well and allowed to adhere under standard culture conditions. After 24 h, cells were then treated with different concentrations of the GH and RH extracts for 24 h. Following treatment, 5 mg/mL MTT solution (3-(4,5-dimethylthiazol-2-yl)-2,5-diphenyltetrazolium bromide; Sigma-Aldrich, St. Louis, MO, USA) prepared in phosphate-buffered saline (PBS) was added to each well, and the plates were incubated for 4 h at 37 °C. The resulting formazan crystals were dissolved in dimethyl sulfoxide (DMSO), and absorbance was recorded at 570 nm using a Multiskan™ FC microplate reader (Thermo Fisher Scientific, Waltham, MA, USA). Cell viability was calculated as a percentage relative to untreated control cells. All experiments were performed in at least three independent biological replicates.

### 2.8. Experiments on Caenorhabditis elegans

#### 2.8.1. *C. elegans* Strains and Growth Conditions

The following *C. elegans* strains were used: wild-type N2 and SJ4103 ZCLS14[*myo-3*::GFP (mit)] (abbr. *myo-3*::GFP), CL2166 (dvIs19 [(pAF15)*gst-4*p::GFP::NLS] III), LD1 (ldIs7 [*skn-1b*/*c*::GFP + rol-6 (su1006)] transgenic strains. The mutant strain QV225 *skn-1*(*zj15*) (abbr. *skn-1*) was also employed. Strains were obtained from the Caenorhabditis Genetics Center (CGC). Nematodes were grown on nematode growth medium (NGM) plates and fed with heat-killed *Escherichia coli* (*E. coli*) OP50 and GH extracts at different concentrations, as indicated. Heat-killed OP50 cells were prepared as follows: bacteria were cultured overnight in Luria Bertani (LB, Sigma-Aldrich International GmbH, Buchs, Switzerland) broth at 37 °C, centrifuged at 6000 rpm for 15 min and suspended in 2 mL of sterile water. Cells were then incubated at 65 °C for 90 min and deposited onto NGM agar plates. Heat-killed cells were also plated on LB agar in parallel to ensure that no viable cells remained.

#### 2.8.2. *C. elegans* Lifespan Assay

Synchronous nematodes were obtained as previously described in Schifano et al., 2021 [20]. Briefly, synchronous nematodes were obtained by placing fertile N2 adults to lay embryos for 8 h on NGM plates, with heat-killed OP50 and GH extracts, or heat-killed OP50 only (as a control), and then sacrificed. When the progeny became fertile (t0), 80 worms per condition were transferred onto new plates seeded with fresh lawns. Lifespan analysis was performed at 16 °C, and worms were daily transferred to new plates and monitored. A worm was considered dead if it did not respond to gentle touch with a platinum wire.

#### 2.8.3. *C. elegans* Healthspan Assays

For healthspan analysis, both pumping activity and locomotion rate were assessed in 1- and 10-day-old adult nematodes.

Pumping rate was measured under an Axiovert 25 microscope (Carl Zeiss, Oberkochen, Germany), by counting the number of grinder contractions of 10 animals for each treatment, for a period of 30 s, as described in Guantario et al., 2018 [21]. Locomotion rate by body bending was quantified by transferring 10 nematodes with a platinum wire into a 50 µL drop of M9 buffer, allowing them to swim freely and monitoring the number of head thrashes within 30 s under an Axiovert 25 microscope (Carl Zeiss, Oberkochen, Germany).

#### 2.8.4. *C. elegans* Intestinal Permeability Assay

Intestinal permeability was assessed in 10-day-old adult nematodes grown on NGM plates seeded with heat-killed *E. coli* OP50 and supplemented with 1 mg/mL of GH. Age-matched worms grown under the same conditions but without extract exposure were used as control. Ten worms per condition were transferred to a microscope slide with 50 µL of M9 buffer and washed several times to remove residual bacteria. Nematodes were then incubated for 3 h in the dark with 100 µL of a 5% Brilliant Blue FCF (Sigma-Aldrich, St. Louis, MO, USA) solution. After the incubation time, worms were washed again with M9 and mounted on 3% agarose pads containing 20 mM sodium azide for fixation. Nematodes were then observed under an Axiovert 25 microscope.

#### 2.8.5. *C. elegans* Fluorescence Analysis of Transgenic Strains

At 1 day, 5 days and 10 days of adulthood, synchronized *myo-3*::GFP, *gst-4*::GFP, *skn-1*::GFP transgenic worms fed heat-killed OP50 and 1 mg/mL of GH were mounted on 3% agarose pads containing 20 mM sodium azide for fixation and then observed using an Axiovert 25 microscope (Carl Zeiss, Oberkochen, Germany). The experiments were repeated three times, and 10 worms per group were used in each experiment. Fluorescence intensity was quantified with ImageJ v. 1.53e. Scale bars were inserted with Zeiss ZEN Microscopy Software 2011. Quantification of *myo-3*::GFP images was performed as described in Li et al., 2026 [22], with slight modifications. Briefly, *myo-3*::GFP synchronized GH-treated worms were observed at 1, 5 and 10 days of adulthood, and a total of 60 to 70 images of body wall muscles between the pharynx and vulva were captured. The images were then categorized into 5 classes ranging from well-preserved and dense round structures (class 1) to more filamentous and sparser ones (class 5). Worms fed only heat-killed OP50 served as control.

#### 2.8.6. *C. elegans* Cytosolic and Mitochondrial Reactive Oxygen Species (ROS) Assays

The levels of cytosolic and mitochondrial ROS were assessed in 1-, 5-, and 10-day-old N2 adult *C. elegans* grown on plates seeded with heat-killed OP50 and supplemented with 1 mg/mL of GH. Worms fed only with heat-killed OP50 served as controls. To evaluate ROS production under oxidative stress conditions, parallel groups of worms (both GH-treated and untreated) were exposed to hydrogen peroxide (H_2_O_2_) prior to fluorescence measurements. Cytosolic ROS production was assessed using the fluorescent probe 2′,7′-dichlorodihydrofluorescein diacetate (H_2_DCF-DA, 287810–100MG, Sigma-Aldrich, St. Louis, MO, USA). Worms were washed with an M9 buffer, and analysis was performed as reported in Ficociello et al. (2023) [23]. Following a 1 h incubation at 20 °C on a shaker in the dark, fluorescence was measured using a multiwell plate reader (Promega GloMax^®^ Multidetection System, Madison, WI, USA) with excitation/emission wavelengths of 485/520 nm. For mitochondrial ROS detection, worms at 1 and 10 days of adulthood were washed with M9 and incubated for 1 h in 5 µM MitoSOX Red^TM^ (Thermo Fisher Scientific Inc., Waltham, MA, USA) at room temperature. After incubation, worms were washed again in M9 buffer, mounted on 3% agarose pads with 20 mM sodium azide and observed under an Axiovert 25 fluorescence microscope (Carl Zeiss, Oberkochen, Germany). Fluorescence intensity was quantified with ImageJ v. 1.53e.

#### 2.8.7. *C. elegans* Real-Time qPCR

RNA of 200 1-day-old adults supplemented with heat-killed OP50 and 1 mg/mL of GH from embryo hatching was extracted using miRNeasy Micro Kit (QIAGEN, Hilden, Germany) and real-time analysis using a Rotor-Gene Q real-time PCR cycler (QIAGEN, Hilden, Germany) was performed according to Schifano et al. (2019) [24]. The selective primers (200 nM) for genes involved in oxidative stress response are reported in Table S1. Quantification was performed using a comparative Ct method (Ct = threshold cycle value). Briefly, the differences between the mean Ct value of each sample and the Ct value of the housekeeping gene (*act-1*) were calculated: Δ_Ctsample_ = C_tsample_ − C_act-1._ The result was determined as 2^−ΔΔCt^, where ΔΔCt = Δ_Ctsample_ − Δ_Ctcontrol_. The experiment was performed in triplicate.

#### 2.8.8. *C. elegans* mtDNA/chrDNA Ratio

The ratio between mtDNA and chrDNA was quantified as previously described in Ficociello et al. (2023), with some additional tuning [23]. To summarize, 25 age-synchronized 1-, 5-, and 10-day-old adult animals grown on heat-killed OP50 and 1 mg/mL of GH were harvested in 90 μL of Standard 1× PCR Buffer in nuclease-free H_2_O supplemented with 1 μg/mL proteinase K in a PCR tube. The worms were immediately frozen at −80 °C and then lysed using a thermocycler (65 °C for 1 h followed by 95 °C for 15 min). The resulting supernatants were collected, and DNA concentration was quantified using a NanoDrop 2000 spectrophotometer (Thermo Fisher Scientific, Waltham, MA, USA). The samples were then diluted to obtain a concentration of 20 ng/μL and used for RT-qPCR to quantify mitochondrial genomic copies of *nd-1* (NADH-ubiquinone oxidoreductase chain 1) and *cox-4* (nuclear DNA coding gene). Relative values of *nd-1* and *cox-4* were compared within each sample to represent the mtDNA per nuclear genome. The forward and reverse primers for *nd-1* and *cox-4* genes are described in Table S1. The experiment was performed in triplicate.

#### 2.8.9. *C. elegans* Oxygen Consumption Rate Assessment

Oxygen consumption measurements were performed according to Ficociello et al. (2023) [23]. To summarize, for each condition, about 1000 L4 or 10-day-old adult worms, treated with heat-killed OP50 and 1 mg/mL of GH from embryo hatching, were washed twice and resuspended in 200 μL of M9 buffer. Worms were then loaded in the Reaction Vessel of a previously calibrated Oxygen Electrode Chamber in which 800 μL of M9 buffer had been already added. Respiration studies were performed using a Clark Oxygen Electrode (Hansatech Instruments, Norfolk, UK). The change in oxygen concentration was recorded over a period of 10 min. The rate of oxygen consumption was then normalized against the total soluble proteins in each sample. The experiments were repeated three times.

#### 2.8.10. *C. elegans* Mitochondrial Function Analysis

MitoTracker^TM^ Red CMXRos in vivo assay was performed as described in Tanaka et al. (2011) [25]. Briefly, 1-, 5-, and 10-day-old adult worms, hatched on NGM agar plates seeded with heat-killed OP50 and 1 mg/mL of GH, were washed with M9 medium and then incubated for 20 h at 20 °C with 150 μL of 5 μM Mitotracker Red CMXRos (Invitrogen, Carlsbad, CA, USA) solution. Following incubation, worms were washed again with M9 and mounted on 3% agarose pads containing 20 mM sodium azide for fixation. Samples were observed using an Axiovert 25 microscope (Carl Zeiss, Oberkochen, Germany) at 5× enhancement, and fluorescence intensity was quantified with ImageJ v. 1.53e. Worms treated only with heat-killed OP50 served as control. The experiment was performed in triplicate.

#### 2.8.11. *C. elegans* Thermal Stress Assays

Thermal resistance assay was performed as described in Zhou et al., 2026 [26]. Briefly, synchronized L4 adult worms were grown on GH-augmented NGM plates until the second day of adulthood. Nematodes were then moved to new plates seeded only with heat-killed *E. coli* and placed in an incubator at 37 °C. Survival of the worms was observed hourly, and each group was composed of 90 worms spread equally between three parallel replicates. Worms fed only heat-killed *E. coli* served as control. The experiment was performed in triplicate.

### 2.9. Spot Test for Assessing Antibacterial Activity of GH Extract

The minimum bactericidal concentration (MBC) was determined from growth on agar using the indicator strains *Pseudomonas aeruginosa* PA01 (*P. aeruginosa*), *Escherichia coli* NCIMB11943 (*E. coli*), *Bacillus cereus* NCIMB9373 (*B. cereus*), and *Staphylococcus aureus* NCDO949 (*S. aureus*), belonging to the culture collection of the Department of Biology and Biotechnologies “C. Darwin” (Sapienza University of Rome, Italy), routinely propagated in Luria Bertani (LB) broth at 37 °C [27]. Bacterial cultures were grown on Mueller–Hinton liquid media overnight and diluted using sterile 0.9% (*w*/*v*) NaCl solution to a concentration of 2 × 10^6^ cells/mL. Pomegranate powder extract (punicalagin 1.5% and ellagic acid 0.017%) was dissolved in distilled water at a starting concentration of 40 mg/mL and serially diluted in Mueller–Hinton media to the different concentrations tested.

In the multi-well plate, each well was filled with 95 µL of serially diluted pomegranate extract, and subsequently, 5 µL of bacterial culture was added to each well to obtain a final concentration of 10^5^ cells/mL. MBC was determined by transferring aliquots from wells on NB agar media and incubated overnight. The growth of bacterial colonies was evaluated after 0, 2 and 21 h of exposure to pomegranate extract.

### 2.10. Statistical Analysis

All experiments were performed in at least three independent replicates. Data are presented as mean ± standard deviation (SD). Statistical significance was assessed using Student’s *t*-test or one-way ANOVA followed by Bonferroni’s post hoc test, using GraphPad Prism 9.0 software (GraphPad Software Inc., La Jolla, CA, USA). Differences were considered statistically significant at *p* < 0.05 and are indicated as follows: * *p* < 0.05, ** *p* < 0.01, and *** *p* < 0.001.

## 3. Results and Discussion

### 3.1. Extraction and Characterization of Pomegranate Peel Extracts

Granato and Roce pomegranate peels were subjected to different extraction methods using environmentally friendly solvents (water, ethanol or hydroalcoholic mixtures, 1 h at room temperature) to optimize the yield of polyphenolic compounds under mild extraction conditions. The extraction conditions were selected based on previous studies demonstrating their effectiveness for recovering pomegranate polyphenols and ellagitannins from pomegranate peel using environmentally friendly extraction procedures [28,29,30]. All extraction parameters (extraction time, temperature, solid-to-solvent ratio, and stirring conditions) were kept constant to enable direct comparison of the extraction efficiency of the different environmentally friendly solvent systems. Therefore, the present study was designed as a comparative evaluation rather than an optimization of the extraction process.

The results showed significant differences, with hydroalcoholic extractions showing the highest yields (27–28%) followed by ethanolic extractions (17–24%) and aqueous extractions (10–11%).

According to the literature, ethanol–water mixtures, due to their intermediate polarity, promote better solubilization and diffusion of phenolic compounds, increasing cell wall disruption and mass transfer, and improving overall extraction efficiency [31,32,33]. The use of hydroalcoholic mixtures is consistent with the principles of green chemistry and circular economy, promoting sustainable processes compatible with nutritional applications [8].

The HPLC-DAD analyses at 360 nm (See Supplementary Materials, Figures S1 and S2), carried out to characterize and quantify the main ellagitannins (punicalin, punicalagin and ellagic acid) in the six pomegranate peel extracts, revealed punicalagin as the predominant compound in all samples.

The total content of punicalagin, as the sum of the α and β anomers, was highest in sample GH (See Supplementary Materials, Table S2), in which about 135 mg/g of dry extract was quantified, and lowest in RA (about 60 mg/g), showing statistically significant differences depending on the solvent used (*p* < 0.05). Punicalin (PUN) showed a very low content, with a maximum level of about 1.6 mg/g, as the sum of anomers in GH, while it was undetectable in aqueous extracts. Ellagic acid (EA) contents showed a maximum value in RH (about 6 mg/g) and a minimum in RA (1.15 mg/g).

Thus, the hydroalcoholic extractions provided the highest extraction yields, with punicalagin (PU) contents of about 3.4 g/100 g of fresh peel of G and 2.9 g/100 g of R and EA contents of about 100 mg/100 g fresh peel of G and 150 mg/100 g fresh peel of R. These findings confirm that ethanol–water mixtures ensure greater extraction efficiency and selectivity toward ellagitannins [34,35].

The PU content (up to 13.5 g/100 g DE) was higher than that reported by Dimitrijević et al. (2024) (5.4 g) [36], but lower than the levels observed by Sabraoui et al. (2020) [35] (21.6 g) using pressurized liquid extraction (PLE). Similarly, EA contents (up to 600 mg/100 g) were below those reported by Benchagra et al. (2021) (2.5 g/100 g DE) [37]. Such differences are likely due to the milder extraction conditions adopted in the present study, based on moderate temperatures and eco-friendly solvents, fully aligned with the principles of green chemistry [1].

Overall, the ethanol–water mixture proved to be an efficient and sustainable solvent system for extracting ellagitannins from pomegranate peels, achieving acceptable yields and favorable selectivity for punicalagin and ellagic acid. The extraction procedure applied in this study provides an environmentally compatible approach that can be easily scaled up for industrial applications. supporting the use of green and low-impact extraction processes for the valorization of agro-industrial by-products, consistent with the broader framework of circular economy and sustainable production [1,8].

### 3.2. Anti-Radical and Antioxidant Activity

The anti-radical and antioxidant activities of pomegranate peel extracts were evaluated by DPPH and FRAP assays. Results are reported in Table S3 (Supplementary Materials) and are expressed as milligrams gallic acid equivalents per gram of dry extract (GAE) for DPPH, and as milligrams ellagic acid equivalents per gram of dry extract (EAE) for FRAP.

For the DPPH assay, all tested extracts confirmed substantial anti-radical activity. The hydroalcoholic extracts (RH and GH) displayed the highest levels of gallic acid equivalents, 186–189 mg/g of extract, ethanolic extracts (RE and GE) showed slightly lower values (about 180 mg/g, but marked differences between R and G cultivar and higher standard deviations were shown), while the aqueous extracts (RA and GA) were much less rich in GAE, (about 78 mg/g) but comparable in EAE of ethanolic extracts. The G cultivar, both in the aqueous and ethanolic extracts, showed higher values of GAE compared with the R cultivar, as well as in the two hydroalcoholic extracts, where the gallic acid equivalent values overlapped.

In the FRAP assay, a similar trend was observed. The RH and GH extracts again exhibited the highest ferric-reducing power, with ellagic acid equivalents of about 50–55 mg/g dry extract, and the ethanolic and aqueous extracts (GA, RA, GE, RE) displayed comparable antioxidant capacities (between 40 and 44 mg/g dry extract). All pomegranate peel extracts possessed antioxidant activity consistent with previous studies [4,38]. Ethanol or hydroalcoholic mixture improves the extraction of antiradical compounds compared with aqueous extraction, by more than 2- or 3-fold; hydroalcoholic mixture even improves the extraction of antioxidant components compared with ethanol, other than relative to water extraction, but only by about 13% more. Overall, although water is an excellent solvent for highly polar compounds, it is less efficient in selectively extracting ellagitannins, an effect already reported in the literature [39].

### 3.3. In Vitro Cytotoxicity on HaCaT Cells

**Figure 1:**
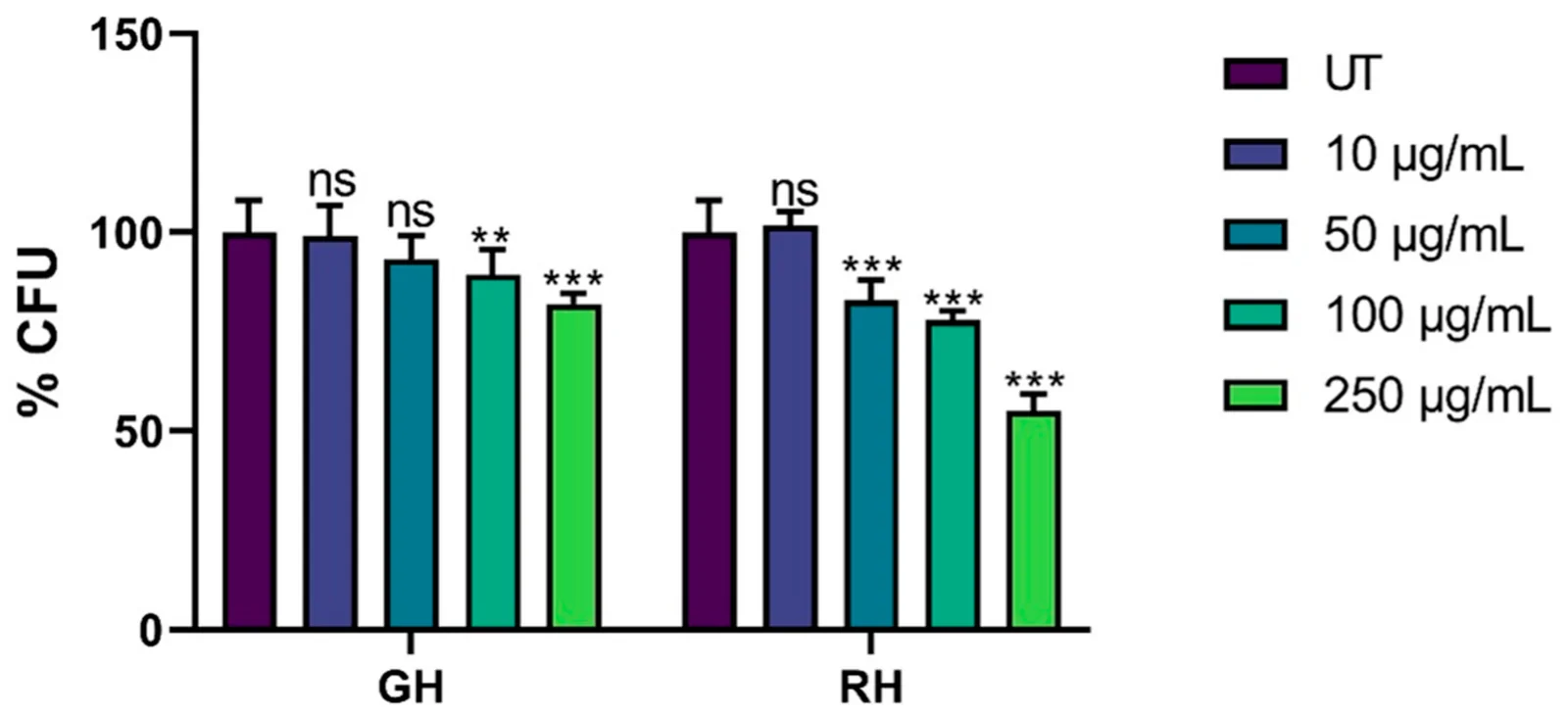
Cell viability of HaCaT cells following 24 h exposure to GH and RH hydroalcoholic extracts (10, 50, 100, and 250 μg/mL), as determined by the MTT assay. Cell viability was expressed as a percentage of the untreated control (UT). Data are presented as mean ± SD from three independent experiments. Statistical analysis was performed using two-way analysis of variance (ANOVA) followed by Bonferroni’s multiple comparisons test (** *p* < 0.01; *** *p* < 0.001; ns: not significant). According to ISO 10993-5, cell viability ≥70% is considered indicative of the absence of cytotoxicity.

Based on the phytochemical characterization and antioxidant evaluation, only the hydroalcoholic extracts (GH and RH) were selected for further biological investigations. This choice was supported by their superior extraction yields, higher ellagitannin content, and stronger antioxidant performance compared with the aqueous and ethanolic extracts. Therefore, the cytotoxicity of the hydroalcoholic extracts was evaluated in HaCaT cells using the MTT assay. Cells were exposed to increasing concentrations (10, 50, 100, and 250 μg/mL) of GH and RH extracts. According to the ISO 10993-5 standard, samples are considered non-cytotoxic when cell viability remains ≥70% that of the untreated control [40]. Moreover, since keratinocytes are the predominant cell type in the epidermis and play an essential role in maintaining skin homeostasis and protecting against oxidative damage, preserving their viability is an essential prerequisite for the development of potential dermatological or cosmetic applications. As shown in Figure 1, GH exhibited a more favorable cytocompatibility profile than RH. Indeed, treatment with GH resulted in cell viabilities of 99%, 93%, 89%, and 81% at 10, 50, 100, and 250 μg/mL, respectively. Since cell viability remained above the 70% threshold at all tested concentrations, GH could be considered non-cytotoxic under the experimental conditions, although a moderate concentration-dependent reduction in viability was observed. In contrast, RH induced a more pronounced reduction in cell viability, resulting in values of 100%, 82%, 77%, and 55% at the corresponding concentrations. While RH remained non-cytotoxic up to 100 μg/mL, exposure to 250 μg/mL reduced cell viability below the 70% threshold, indicating a cytotoxic effect at this concentration. Considering its more favorable cytocompatibility profile, together with its phytochemical composition and its in vitro antioxidant and antiradical activities, GH was selected for subsequent in vivo investigations using the model organism *Caenorhabditis elegans*.

### 3.4. Effects of Hydroalcoholic Extract GH on Caenorhabditis elegans Lifespan

Following the preliminary in vitro screening, GH was selected for subsequent in vivo evaluation in *C. elegans* as the extract showing the most favorable overall profile. Compared with RH, GH combined the highest ellagitannin content with superior antioxidant activity and absence of cytotoxicity across the tested concentration range. Moreover, punicalagin accounted for approximately 95% of the quantified ellagitannin-related compounds, making GH a representative ellagitannin-rich extract for investigating the biological effects of pomegranate peel phytochemicals, while not excluding possible synergistic contributions from the remaining constituents [41].

**Figure 2:**
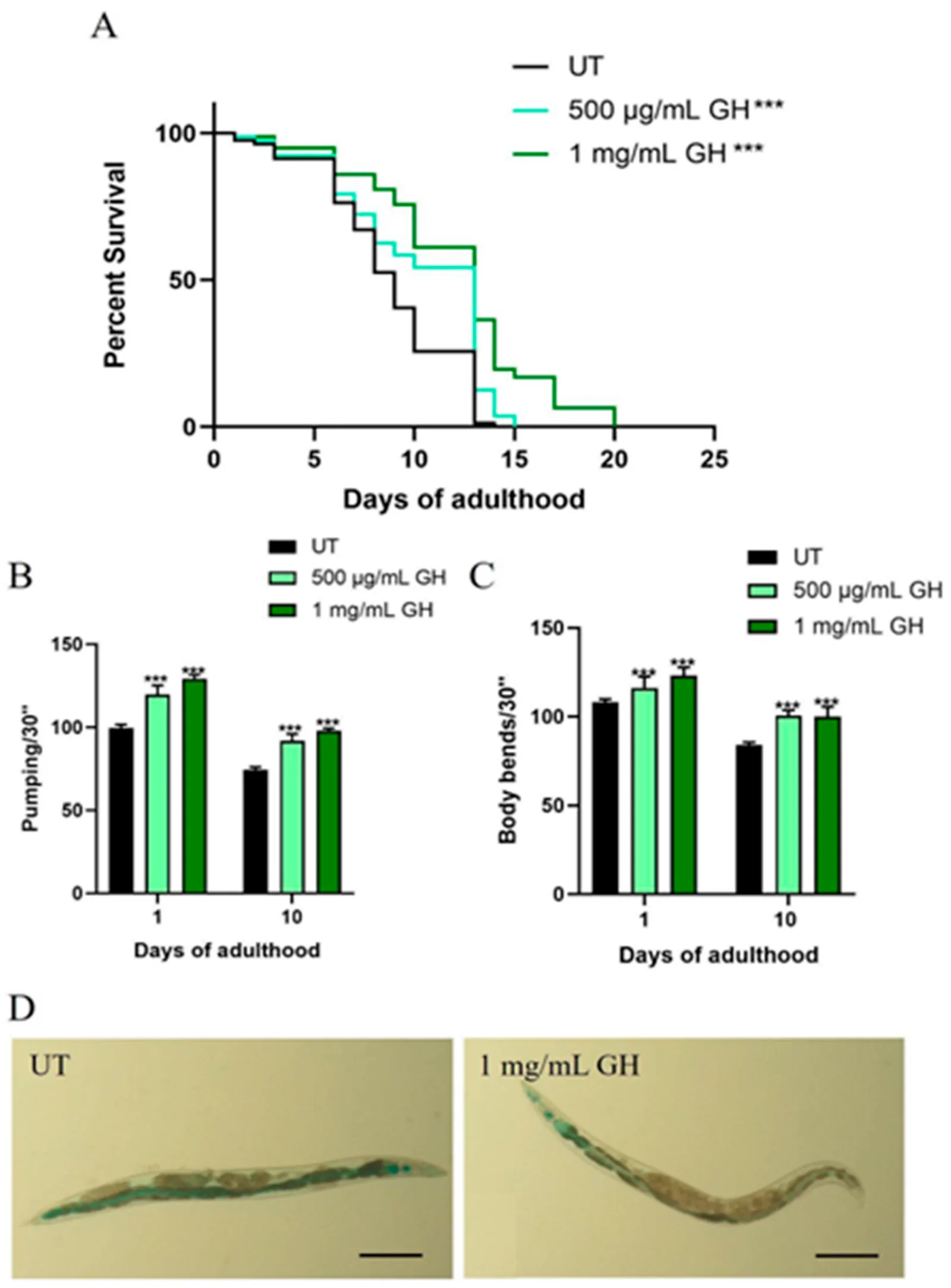
Impact of GH extract on *C. elegans* viability and healthspan. (**A**) Kaplan–Meier survival plot of N2 wild-type *C. elegans* worms fed with heat-killed *E. coli* OP50 and supplemented with 500 µg/mL or 1 mg/mL GH. Worms fed with OP50 alone (untreated, UT) were used as reference control. Data are presented as a combined survival analysis from three independent experiments (in triplicate). A total of *n* = 80 worms were analyzed for each condition per single experiment. Statistical differences between GH-treated groups and the untreated control group were determined using the Log-Rank (Mantel–Cox) test (*** *p* < 0.001). (**B**) Pharyngeal pumping contractions and (**C**) locomotion ability of N2 wild-type worms were measured at different developmental stages following GH treatment initiated from embryo hatching. The aging markers were assessed over a 30 s interval. Data represent the mean pharyngeal contractions and body thrashes quantified from 10 individual worms for each experimental condition. Untreated worms served as the reference control (UT). Data are presented as mean ± SD. Statistical analysis was performed by two-way ANOVA with the Bonferroni post-test; asterisks indicate significant differences (*** *p* < 0.001). (**D**) Effects of GH on *C. elegans* intestinal permeability. FCF Brilliant Blue staining in 10-day-old adult worms treated with GH at the concentration of 1 mg/mL, compared to untreated worms (UT). Scale bar: 100 μm.

To evaluate the potential pro-longevity effects of the GH extract, survival curves of N2 worms were generated across a concentration range from 10 µg/mL to 20 mg/mL (Table S4; Figure 2A). No significant differences in lifespan were observed at the lowest doses (10–100 µg/mL). In contrast, worms supplemented with 500 µg/mL or 1 mg/mL GH showed a clear, dose-dependent extension of survival (Figure 2A). In both groups, 50% survival was reached on day 13, whereas untreated controls reached the same threshold on approximately day 10. Conversely, increasing the concentration above 1 mg/mL did not further improve survival, and treatment with 20 mg/mL GH resulted in a marked reduction of 38% in lifespan, indicating a clear toxicity at this dose (Table S4). Overall, these data show that GH exerts beneficial effects on longevity within a defined dose window, with 1 mg/mL emerging as the most effective non-toxic concentration. It should be noted that the concentrations used in HaCaT cells and *C. elegans* are not directly comparable. In HaCaT monolayer cultures, cells are directly exposed to the nominal concentration of the extract present in the culture medium. In contrast, *C. elegans* is a whole-organism model in which bioactive compounds must be ingested, absorbed through the intestinal tract, metabolized, and distributed to target tissues, resulting in lower effective intracellular exposure. Consequently, higher nominal concentrations are commonly required in vivo to achieve biologically relevant effects.

The dose-dependent nature of GH’s effects deserves consideration. The biphasic dose–response observed is consistent with a hormetic mechanism, a phenomenon frequently described for phytochemical-rich plant extracts, whereby low or moderate doses activate adaptive stress-response pathways that promote cellular resilience and longevity, whereas excessive concentrations lose their beneficial effects or become detrimental [42]. Accordingly, the beneficial effects observed at 500 μg/mL and 1 mg/mL likely reflect activation of protective cellular responses rather than a simple dose-dependent antioxidant effect. Previous studies with pomegranate extracts have similarly reported opposing outcomes depending on dosage, with low concentrations promoting longevity and high concentrations reducing survival [43,44]. This biphasic response is characteristic of hormesis and underscores the importance of identifying optimal dosing windows for nutraceutical applications.

Although the uptake and metabolism of individual ellagitannins were not directly investigated in the present study, these compounds are generally characterized by limited bioavailability because of their high molecular weight and complex chemical structure, which restrict intestinal absorption [45]. In mammals, ellagitannins are hydrolyzed to ellagic acid and subsequently converted by the gut microbiota into urolithins, metabolites considered major contributors to their biological activity [45,46]. By contrast, the metabolic fate of ellagitannins in *C. elegans* remains poorly understood. Since worms were continuously exposed to GH from embryo hatching throughout adulthood, the observed lifespan extension most likely reflects the cumulative effects of long-term dietary supplementation rather than an acute pharmacological response.

Lifespan extension is commonly associated with a delay in the onset and progression of age-related functional decline [47]. For this specific reason, different aging markers, such as pharyngeal pumping rate and body bending of N2 wild-type worms treated with different concentrations of GH, were assessed at 1 and 10 days of adulthood (Figure 2B). Pumping rate is defined as the frequency of pharyngeal contractions, which reflects the feeding efficiency of nematodes, while body bending, consisting of the frequency of head-thrashes, indicates the worms’ motility. Both markers decline during the aging process and were therefore used as senescence markers [48,49]. On day 1, worms treated with 500 µg/mL and 1 mg/mL GH exhibited an increase in pumping rate of about 20% and 30%, respectively, compared with untreated controls (Figure 2B). This beneficial effect persisted on day 10, where treated worms maintained pumping rates 20% and 30% higher than those of controls, indicating a delay in the age-related decline of feeding capacity. Locomotion followed a similar pattern: GH supplementation slightly increased body bending frequency on day 1 by 10% at 500 µg/mL and 15% at 1 mg/mL, while at day 10, treated worms still displayed motility values about 15% greater than those of the control group (Figure 2C). Together, these findings show that GH improves neuromuscular performance throughout aging. Another aging marker analyzed in this study was intestinal permeability, since it tends to increase with age in *C. elegans*, as shown in Dambroise et al., (2016) [50]. To visualize any alteration in the integrity of the intestinal tract, Brilliant Blue FCF dye was used, since it diffuses outside of the intestine only if tissue damage is present (Figure 2D). Clear differences emerged by day 10, when control worms exhibited a high proportion of individuals with increased dye penetration, whereas GH-treated worms showed a markedly reduced frequency of permeability defects. These results indicate that GH helps preserve intestinal barrier integrity during aging. Since exposure to GH yielded the most favorable results at the concentration of 1 mg/mL, this was therefore selected as the optimal concentration and used for all subsequent experiments.

**Figure 3:**
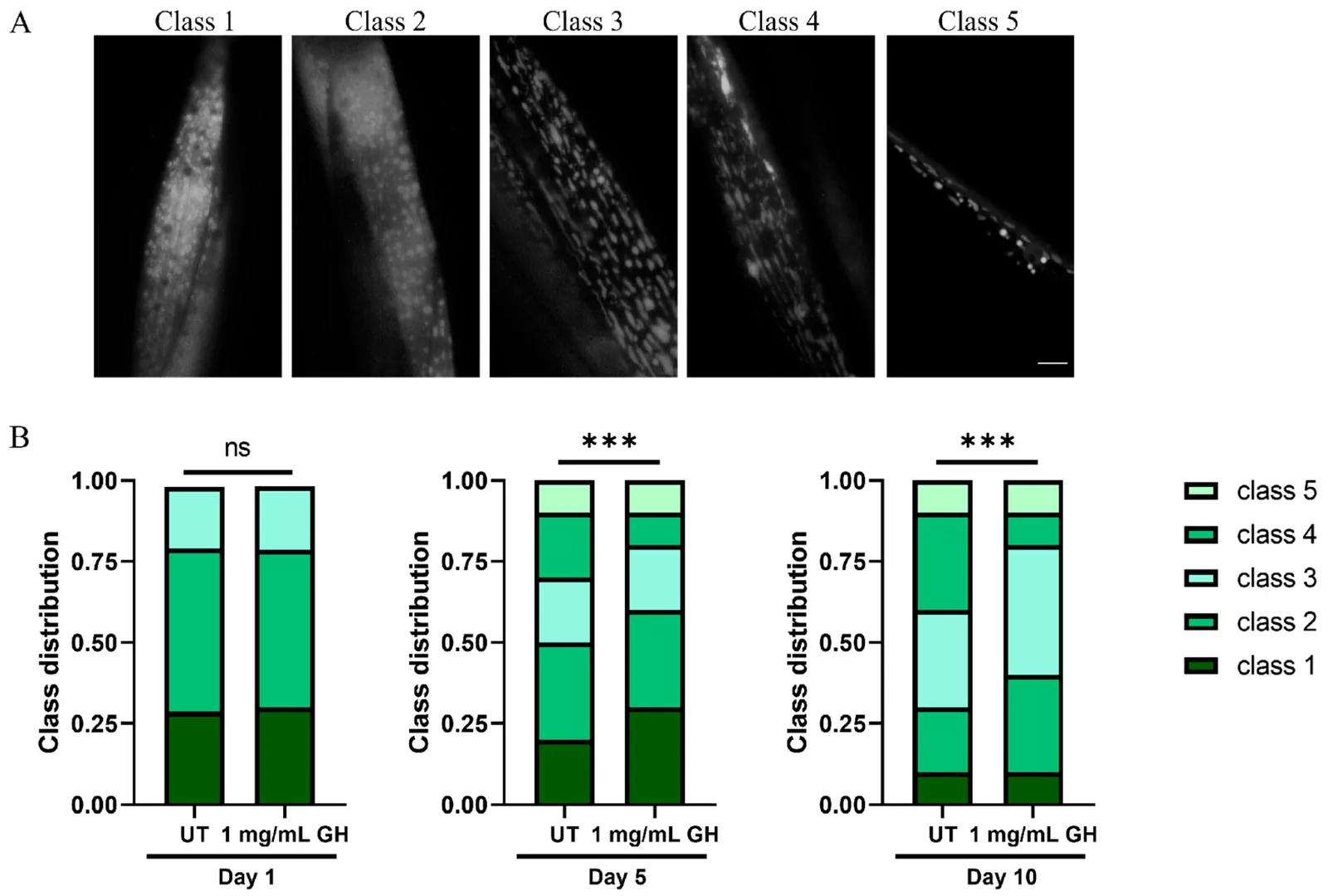
Analysis of muscle tissue integrity using the transgenic *myo-3*::GFP strain. (**A**) Representative fluorescence microscopy images of the five classes of body wall muscle in transgenic worms expressing Green Fluorescent Protein (GFP) specifically in the body wall muscle cells. (**B**) Class distribution of wall muscle structure of *myo-3*::GFP worms supplemented with 1 mg/mL GH from embryo hatching. Nematodes were observed at 1, 5 and 10 days of adulthood. Untreated worms were used as the control group (UT). Scale bar: 10 μm. Statistical analysis was performed using two-way analysis of variance (ANOVA) followed by Bonferroni’s multiple comparisons test (*** *p* < 0.001; ns: not significant).

Since nematodes treated with GH extract showed an increase in motility compared to the control, the muscle structure of worms was observed during the aging process. This was achieved using a transgenic strain of *C. elegans*, *myo-3*::GFP, which allowed the visualization of myosin fibers under fluorescent microscopy. Their morphology transforms from round to a more filamentous one as they degrade with age. As illustrated in Figure 3, while no significant difference was observed between 1-day-old adult treated nematodes and control, this became more evident at 5 and 10 days of adulthood, when treated worms appeared to maintain a more organized filamentous muscle architecture compared to controls.

In summary, GH supplementation significantly improved pharyngeal pumping, locomotion, muscle integrity, and intestinal barrier function during aging. These functional benefits indicate a preservation of neuromuscular and intestinal homeostasis, which are recognized hallmarks of healthspan extension in *C. elegans* [48]. Comparable protective effects on locomotion and muscle structure have been described for pomegranate leaf extracts, which were linked to improved mitochondrial fitness and stress resilience [9]. A similar protective effect was observed at the level of intestinal permeability. As described above, aged GH-treated worms exhibited significantly reduced intestinal permeability, with a lower incidence of the Smurf phenotype. The preservation of barrier function, together with the maintenance of muscle organization and motor activity, indicates that GH confers global protection against the age-associated functional decline of multiple tissues, thus extending not only lifespan but also the period of healthy physiological performance.

### 3.5. Oxidative Stress Analysis in Nematodes Treated with 1 mg/mL of GH Extract

Since oxidative stress plays an important role in the aging process [50], and GH extract showed strong antioxidant activity in vitro, the next step was to explore its in vivo effects on the oxidative stress responses in nematodes.

**Figure 4:**
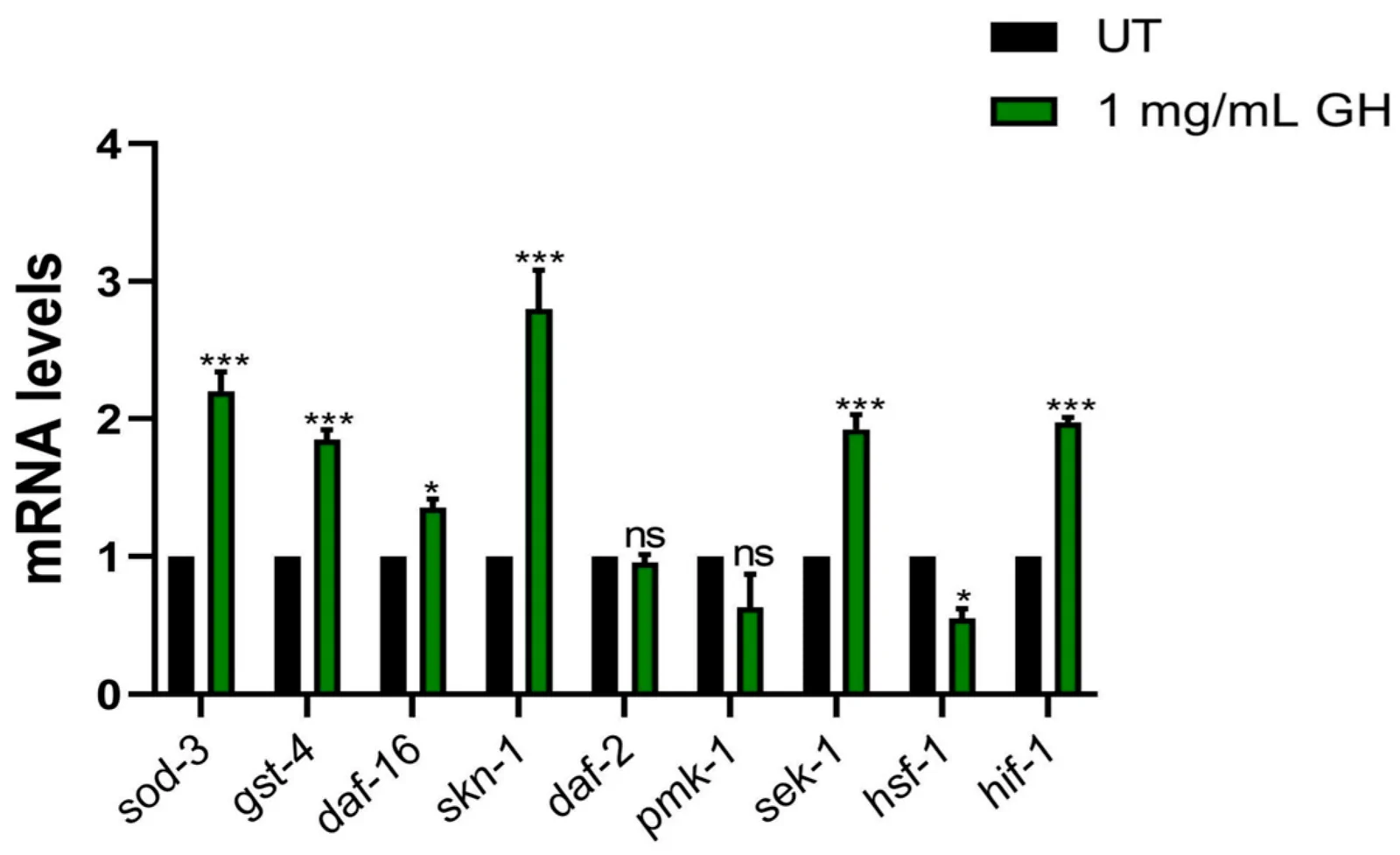
Impact of GH on the expression of oxidative stress-related genes in *C. elegans*. Quantitative real-time PCR (RT-qPCR) analysis. Histograms illustrate the relative mRNA expression levels of different genes within N2 wild-type worms. Worms were treated or not treated with GH starting from embryo hatching and analyzed at the one-day adult stage. Untreated worms served as the control group (UT). Gene expression data were normalized to a reference gene (*act-1*). Experiments were performed in triplicate. Statistical analysis was performed by one-way ANOVA with the Bonferroni post-test; asterisks indicate significant differences (* *p* < 0.05; *** *p* < 0.001; ns: not significant).

Given that pro-longevity and oxidative stress responses in nematodes are mediated by the activation of several conserved signaling pathways, including DAF-2/DAF-16, HSF-1-dependent heat-shock responses, and MAPK-regulated stress pathway, a real-time PCR analysis was performed to investigate the molecular mechanisms potentially triggered by GH supplementation (Figure 4). The transcripts analyzed included those encoding the insulin/IGF-1 receptor DAF-2, the forkhead box O transcription factor DAF-16, the detoxification enzymes superoxide dismutase 3 (SOD-3) and glutathione S-transferase 4 (GST-4), the oxidative-stress-responsive transcription factor SKN-1 (the functional orthologue of human NRF-2), as well as the heat-shock transcription factor HSF-1 and the hypoxia-induced transcriptional regulator HIF-1.

At the molecular level, GH treatment induced the activation of stress-response pathways involved in detoxification and antioxidant defense (Figure 4). RT-qPCR analysis revealed increased transcription of *gst-4*, *sod-3*, and *skn-1*, with expression levels rising by 2-fold, 2.5-fold, and 3-fold, respectively, compared with untreated controls. Mild upregulation of *daf-16* and *hsf-1* was also observed, of about 40% and 50%, respectively, while *daf-2* and *hsf-1* expression remained unchanged. Consistently, fluorescence analysis of the transgenic *skn-1::GFP* strain supported GH-mediated activation of SKN-1 (Figure S5).

**Figure 5:**
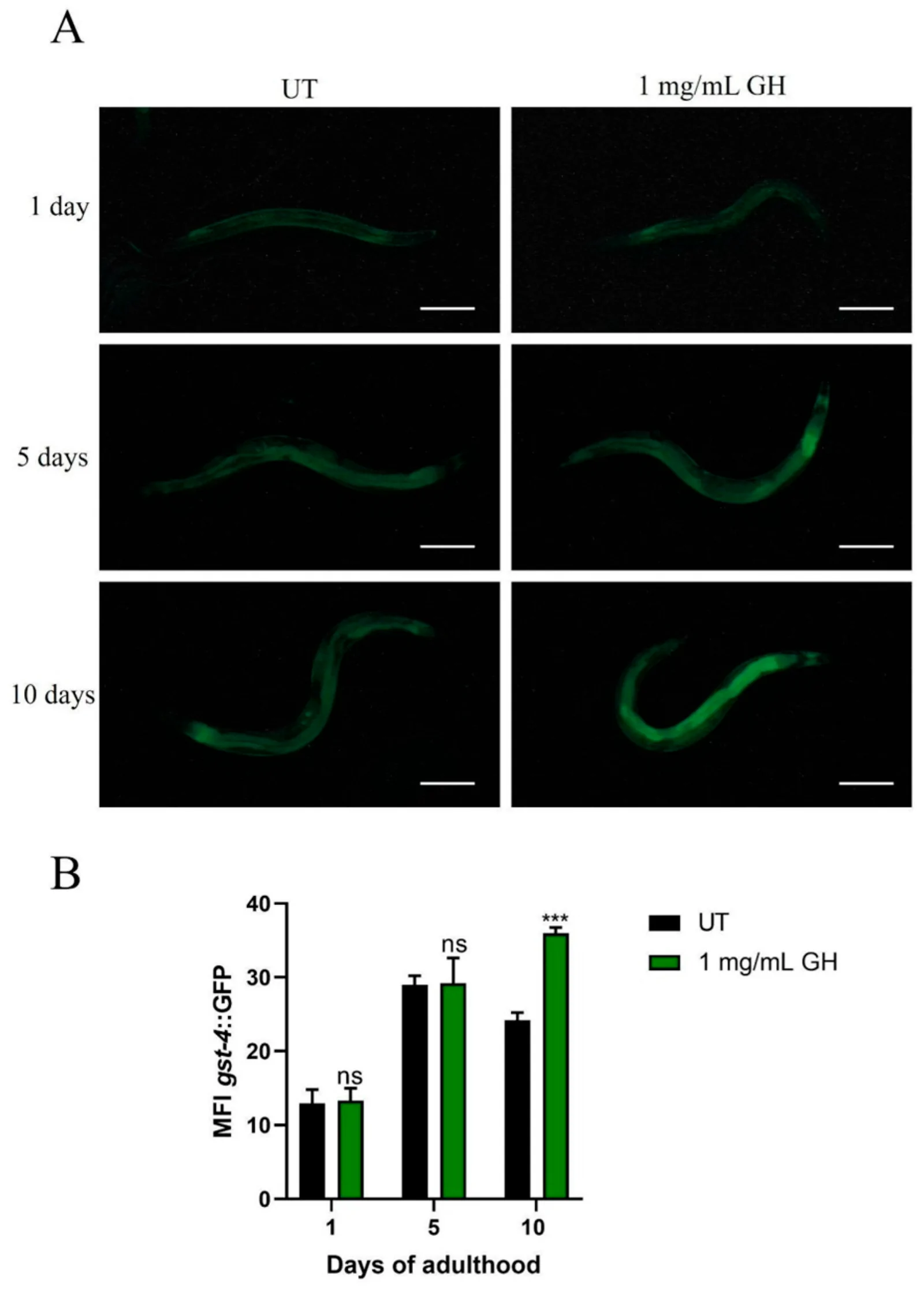
Effect of GH on GST-4 detoxifying enzyme in transgenic *C. elegans* strains. (**A**) Representative fluorescence microscopy images of the *gst-4*:: GFP worm strain at 1, 5 and 10 days of adulthood, after supplementation with 1 mg/mL GH from embryo hatching; (**B**) the corresponding Mean Fluorescence Intensity (MFI) quantification. Untreated worms served as the control group in all assays (UT). Scale bar: 100 μm. Statistical analysis was evaluated using two-way ANOVA followed by the Bonferroni post-test. Data bars represent the mean from three independent experiments, and asterisks denote significant differences (*** *p* < 0.001, ns: not significant) compared to the untreated control.

**Figure 6:**
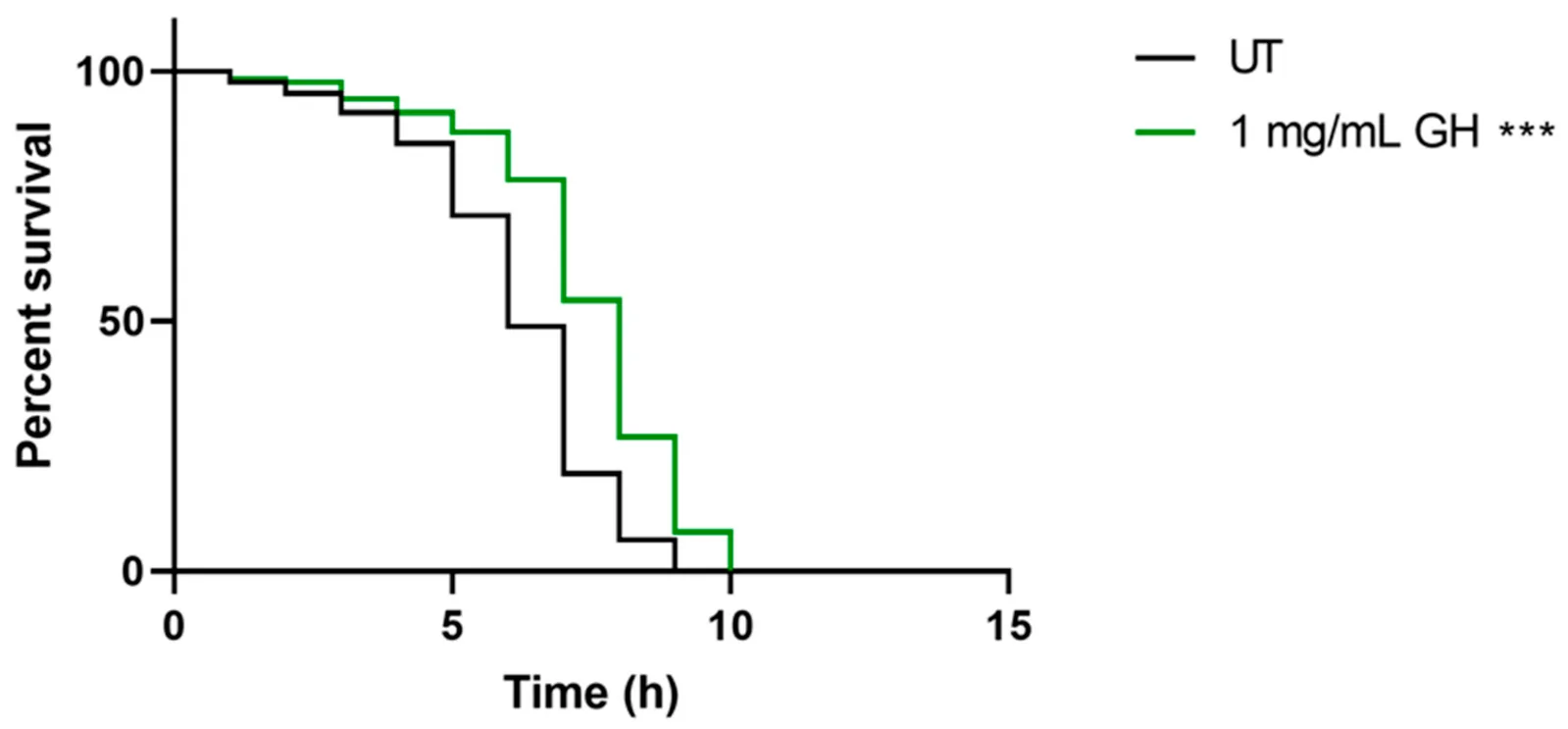
Effect of GH on resistance to acute thermal stress. Survival plot of N2 wild-type *C. elegans* worms fed with heat-killed *E. coli* OP50 and supplemented with 1 mg/mL GH and exposed to thermal stress at 37 °C. Worms fed with heat-killed OP50 alone (UT) were used as control. Data are presented as a combined survival analysis from three independent experiments. Ninety worms were analyzed for each condition per single experiment. Statistical differences between GH-treated nematodes and the untreated control group were determined using the Log-Rank (Mantel–Cox) test (*** *p* < 0.001).

**Figure 7:**
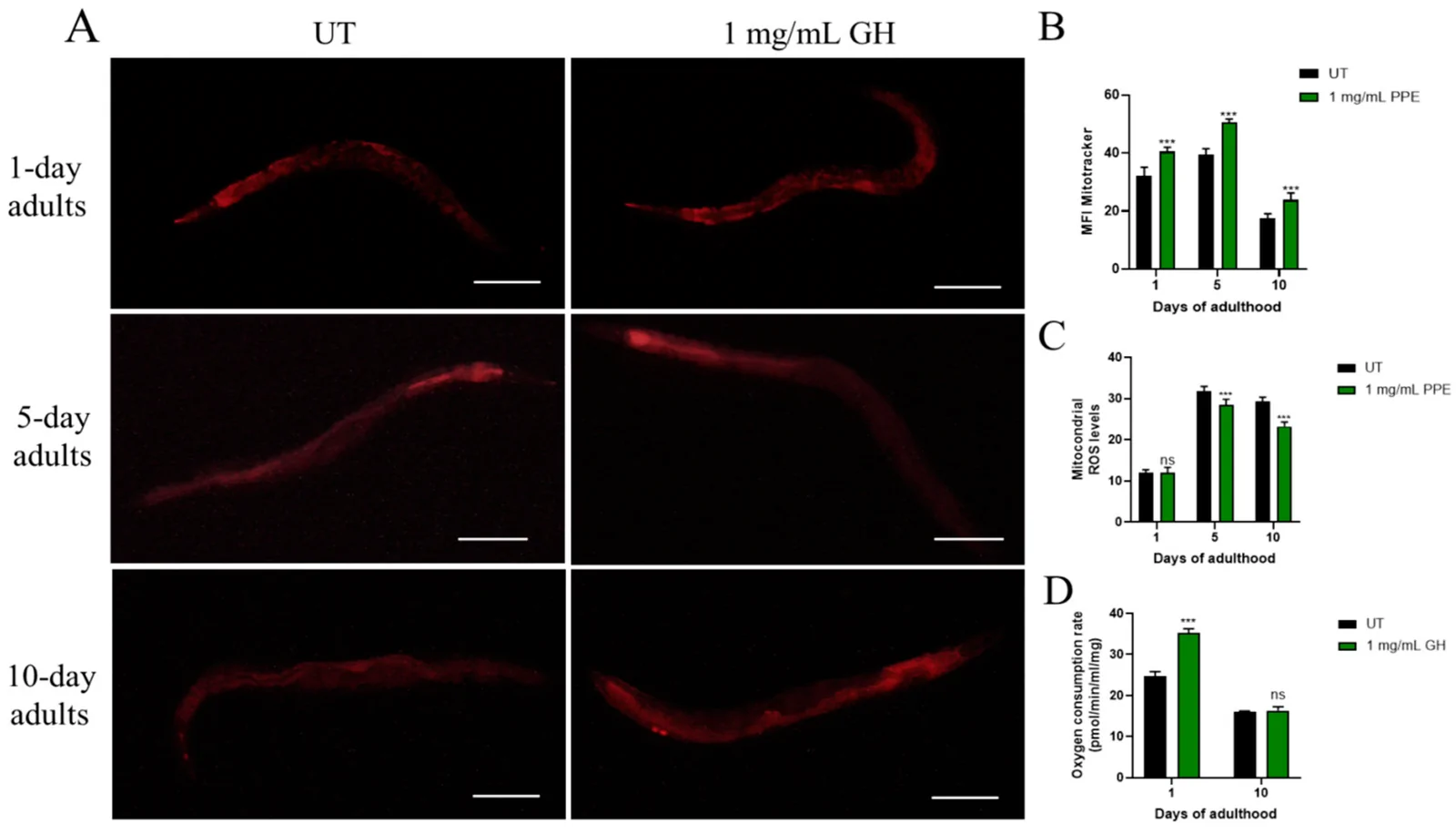
Assessment of mitochondrial function in GH-treated worms. (**A**) Representative fluorescence images and (**B**) quantification of mitochondrial membrane potential in 1-, 5-, and 10-day-old adults, compared to the untreated control group (UT). Scale bar: 100 μm. (**C**) Mitochondrial ROS levels in 1-, 5- or 10-day-old adults treated with 1 mg/mL GH, compared to the untreated control group (UT). (**D**) Oxygen consumption rate was measured in 1- and 10-day-old adult nematodes treated with 1 mg/mL of GH extract, while untreated worms (UT) served as the control group. Respiration rates were normalized to total soluble protein content for each sample. Statistical analysis was evaluated by two-way ANOVA with the Bonferroni post-test (*** *p* < 0.001; ns: not significant). Bars represent the mean of three independent experiments.

The upregulation of the detoxifying enzyme *gst-4* transcript was observed in vivo using the transgenic *gst-4*::GFP strain, which showed increased fluorescence at 10 days of adulthood (Figure 5). The analysis of reactive oxygen species (ROS) in the cytosol was conducted on 1- and 10-day-old worms but did not report any meaningful difference between GH-treated nematodes and the control condition (Figure S3). Since oxidative stress can have interplay with other abiotic stress [51], we decided to perform a thermal resistance assay (Figure 6). We observed that GH increased the maximum survival of treated worms by 1 h when compared to that of the untreated nematodes, which peaked at 9 h. We then decided to further investigate mitochondrial function and oxidative status since GST-4 is involved in both cytosolic and mitochondrial homeostasis [52]. Mitochondrial membrane potential was first assessed using the fluorescent dye MitoTracker Red CMXRos™, which accumulates in active mitochondria in a membrane potential-dependent manner. As shown in Figure 7A,B, GH-treated nematodes displayed a significantly higher MitoTracker fluorescence compared to controls, with an approximately constant 33% increase observed at 1, 5, and 10 days of adulthood. Mitochondrial oxidative stress levels were subsequently evaluated using the fluorophore MitoSOX Red™, which is selectively oxidized by mitochondrial superoxide. As reported in Figure 7C, fluorescence levels were comparable between GH-treated and control worms at 1 day of adulthood, whereas a significant reduction in mitochondrial superoxide accumulation was observed in treated nematodes at 5 days and more prominently at 10 days of adulthood. To further characterize the effects of GH extract on mitochondrial metabolism, oxygen consumption rate was measured in treated and untreated nematodes at 1 and 10 days of adulthood using a Clark-type oxygen electrode. As shown in Figure 7D, young adult worms exposed to 1 mg/mL GH extract exhibited an approximately 40% increase in oxygen consumption rate compared to the control group, while no significant differences were detected between treated and untreated nematodes during aging.

In parallel, mitochondrial content was evaluated by measuring the relative mitochondrial DNA to chromosomal DNA (mtDNA/chrDNA) ratio. As reported in Figure S4, GH supplementation did not induce significant changes in the mtDNA/chrDNA ratio at 1, 5, or 10 days of adulthood compared to controls, indicating that mitochondrial DNA copy number remained unchanged across the analyzed time points.

Altogether, these effects suggest that GH supports mitochondrial integrity and reduces age-related mitochondrial dysfunction, an interpretation consistent with the anti-oxidant properties of ellagitannins and with recent findings that ellagic acid enhances stress resistance in *C. elegans* through DAF-16 activation [9,53,54]. At the molecular level, GH activated multiple conserved stress-response pathways. The upregulation of *gst-4*, *sod-3*, and *skn-1*, together with increased *gst-4*::GFP fluorescence and SKN-1 nuclear localization, indicates stimulation of the SKN-1/NRF2 axis, central to detoxification and oxidative-stress management. Concomitant induction of *daf-16* and the preservation of physiological functions suggest engagement of the insulin/IGF-1 signaling pathway, a primary regulator of longevity in nematodes [55]. Notably, our data also align with emerging evidence highlighting the involvement of HSF-1 and HIF-1 in the anti-aging effects of pomegranate-derived extracts [9]. Given the preservation of muscle structure and improvements in mitochondrial function observed here, similar regulatory mechanisms may underline the biological activity of GH. The convergence of DAF-16, SKN-1, HSF-1, and HIF-1 activity points to a pleiotropic action typical of complex polyphenolic mixtures, where multiple pathways are simultaneously engaged to promote cellular robustness and delay functional decline.

Moreover, O_2_ consumption data suggest that GH supplementation induces an early metabolic adaptation rather than a sustained increase in basal respiration. The transient elevation of oxygen consumption observed in young worms reflects enhanced mitochondrial respiratory activity and metabolic flexibility, rather than mitochondrial overactivation. Similar age-dependent increases in oxygen consumption have been reported for polyphenol-rich extracts and dietary interventions that improve mitochondrial efficiency during early adulthood without accelerating metabolic aging [38,45]. This transient metabolic response is consistent with a hormetic mechanism, whereby moderate and temporary stimulation of mitochondrial respiration supports long-term mitochondrial quality control and functional maintenance.

### 3.6. SKN-1-Dependent Stress Response Mediated by GH Supplementation

**Figure 8:**
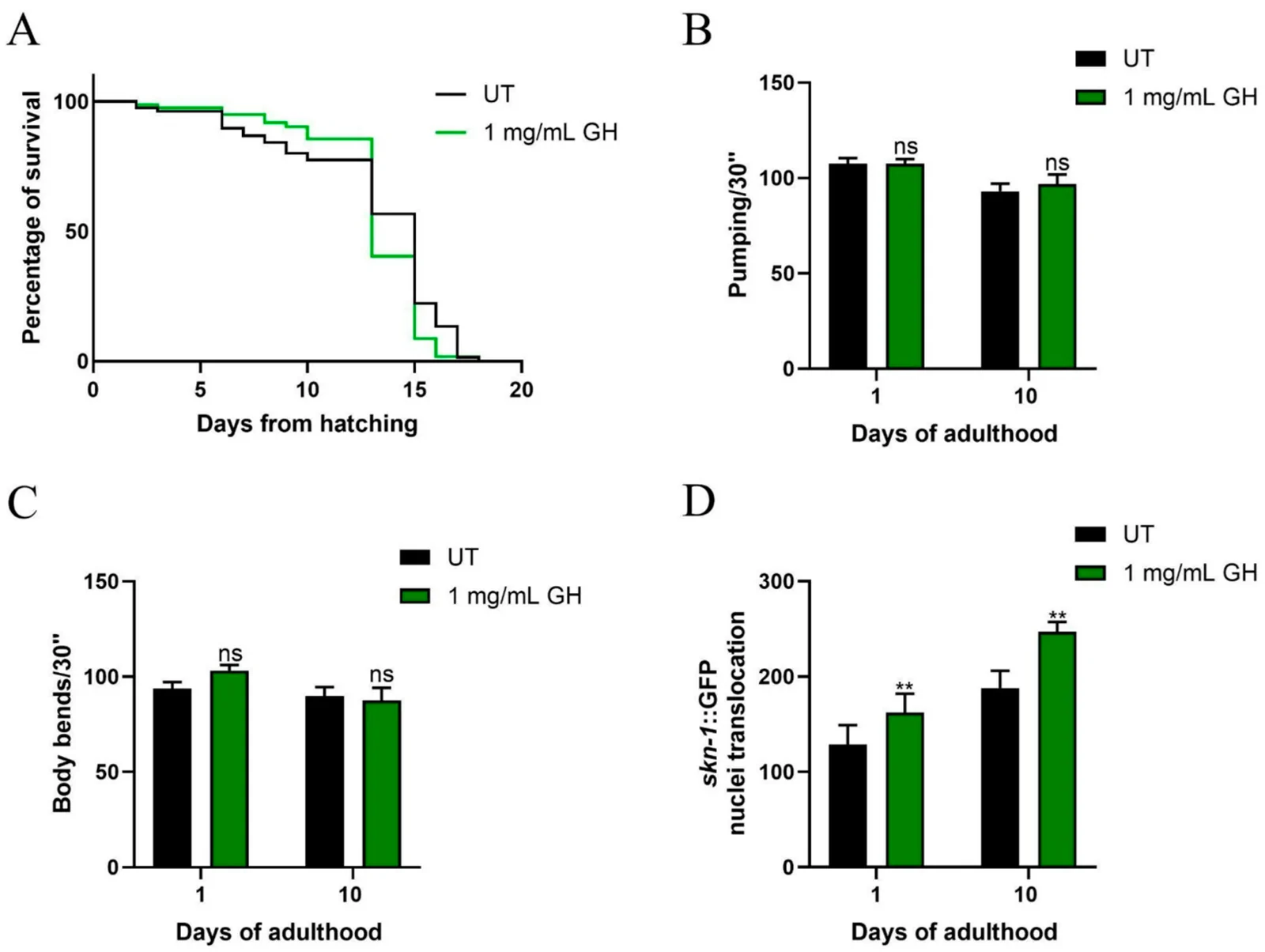
Effect of GH on transcription factor SKN-1 in *C. elegans* mutant strains. (**A**) Kaplan–Meier survival plot of *skn-1C. elegans* mutant worms fed with heat-killed *E. coli* OP50 and supplemented with 1 mg/mL GH. Worms fed with OP50 alone (untreated, UT) were used as reference control. Data are presented as a combined survival analysis from three independent experiments (triplicates). *n* = 80 worms were analyzed for each condition per single experiment. Statistical differences between GH-treated group and the untreated control group were determined using the Log-Rank (Mantel–Cox) test (ns: not significant). (**B**) Pharyngeal pumping contractions and (**C**) locomotion ability of *skn-1* mutant worms measured at different developmental stages following GH treatment initiated from embryo hatching. The aging markers were assessed over a 30 s interval. Data represent the mean pharyngeal contractions and body thrashes quantified from 10 individual worms for each experimental condition. Worms fed with heat-killed OP50 without GH supplementation served as the reference control (UT). (**D**) Quantification of SKN-1 nuclear localization in the *skn-1*::GFP worm strain at 1 and 10 days of adulthood after supplementation with 1 mg/mL GH from embryo hatching. Data are presented as mean ± SD. Statistical analysis was performed by two-way ANOVA with the Bonferroni post-test; asterisks indicate significant differences (** *p* < 0.01; ns: not significant).

The SKN-1 transcription factor, involved in the p38 MAPK pathway, is known to participate in pro-longevity effects [50]. To better assess SKN-1’s role in the manifestation of the observed phenotypes induced by GH extract, *C. elegans skn-1* mutant strain was employed. As illustrated in Figure 8A–C, GH supplementation to *skn-1*-deficient nematodes did not induce any significant improvement in lifespan or in the analyzed healthspan parameters, including locomotion and pharyngeal pumping, compared to the control group. Together, these findings indicate that the beneficial effects of GH extract on longevity and healthspan are dependent on SKN-1 activity.

Moreover, to further strengthen the role of this transcription factor, a *skn-1*::GFP transgenic strain was used to perform a nuclear translocation analysis of SKN-1, to quantify its activation levels (Figure 8D and Figure S3). In both 1- and 5-day-old adult worms, GH-treated nematodes showed a significant increase of 25% and 33%, respectively, in the translocation of SKN-1 compared to the control.

**Figure 9:**
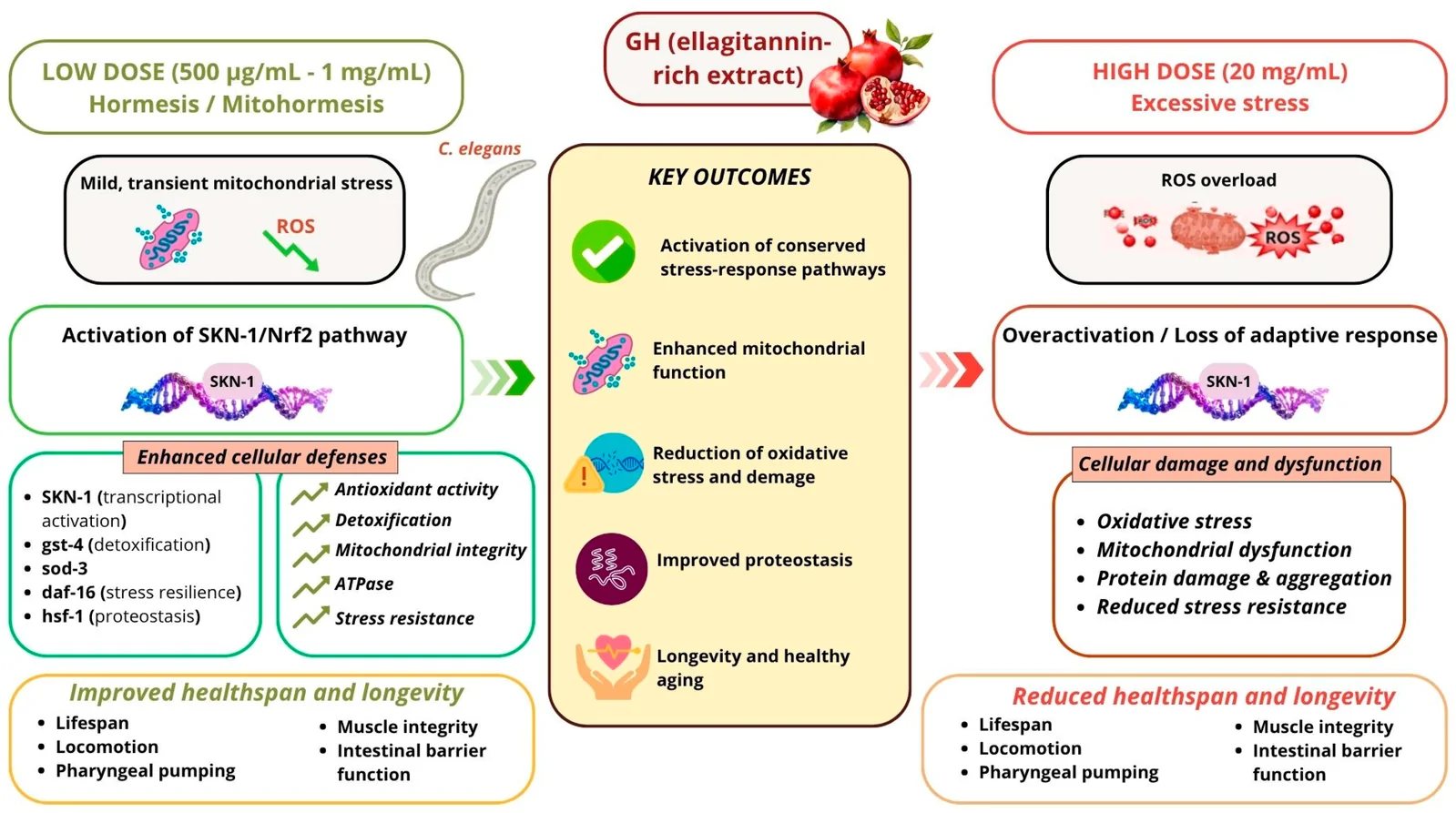
Hormetic and mitohormetic mechanism of ellagitannin-rich extract (GH) in *C. elegans*; dose-dependent activation of stress-response pathways to promote stress resistance, mitochondrial integrity and longevity. Green arrows indicate the beneficial adaptive response associated with low-to-moderate GH concentrations, whereas red arrows indicate the transition toward detrimental effects associated with high GH concentrations.

Collectively, these findings demonstrate that GH-induced improvements in lifespan and healthspan in wild-type worms are mediated by SKN-1, supporting the hypothesis that activation of conserved stress-response pathways underlies the biological activity of the extract. In this regard, the activation of SKN-1 observed in the present study, together with the induction of antioxidant defenses and the improvement of mitochondrial function, supports the hypothesis that ellagitannin-rich pomegranate peel extracts act as hormetic modulators of stress adaptation (Figure 9). Similar mechanisms have been described for several functional food-derived polyphenols, which activate NRF2/SKN-1 signaling, enhance mitochondrial homeostasis, and promote healthy aging through adaptive stress responses rather than through simple radical scavenging activity [42,43].

Although the uptake and metabolism of individual ellagitannins were not directly assessed in the present study, the activation of SKN-1-dependent pathways, together with the increased expression of antioxidant response genes and the improved physiological performance of treated worms, provides indirect evidence that bioactive constituents of the GH extract reached biologically relevant targets. These findings suggest that the extract, or its metabolites generated within the nematode, were sufficiently bioavailable to trigger conserved stress-response mechanisms associated with longevity and healthspan.

From a mechanistic standpoint, SKN-1 represents the functional ortholog of mammalian NRF2 and orchestrates the transcription of genes involved in phase II detoxification, redox homeostasis, and cellular defense [56]. Activation of the SKN-1/NRF2 axis has been repeatedly associated with lifespan extension and increased stress resistance in *C. elegans*, particularly in response to dietary polyphenols and plant-derived bioactive compounds [9,55]. In this context, the increased nuclear translocation of SKN-1 observed upon GH supplementation is consistent with previous reports showing that pomegranate-derived ellagitannins and related phenolics can activate conserved antioxidant signaling pathways rather than acting solely as direct radical scavengers. Notably, the lack of lifespan and healthspan benefits in *skn-1-*deficient mutants indicates that SKN-1 activation is required for the downstream physiological effects induced by GH. Similarly, SKN-1-dependent responses have been described for other nutraceutical extracts, reinforcing the concept that mild activation of stress-response pathways elicits adaptive responses that ultimately enhance organismal resilience and longevity [47].

### 3.7. Effects of the Extracts on Microbial Growth

The antimicrobial effects of punicalagin and ellagic acid are controversial, as some studies have shown that both purified punicalagin and ellagic acid have antimicrobial effects [57,58], while in other studies, these compounds appeared ineffective [59].

The bacterial strains selected for this study were chosen because of their relevance to both public health and the potential cosmetic application of pomegranate peel extracts. *Staphylococcus aureus* is one of the main opportunistic pathogens associated with secondary infections of damaged or compromised skin, whereas *Bacillus cereus* is a ubiquitous spore-forming bacterium frequently found as an environmental contaminant of plant-derived raw materials and cosmetic ingredients [60]. Evaluating the activity of the extracts against these microorganisms therefore provides preliminary information on their potential to contribute both to skin protection and to the microbiological safety of plant-based formulations.

We carried out a spot assay (Figure S6), which demonstrated that both ellagitannin-rich extracts exerted potent antibacterial activity against the Gram-positive pathogens *Bacillus cereus* and *Staphylococcus aureus*. Both extracts exhibited a concentration- and time-dependent inhibitory effect against the two bacterial species. Immediately after treatment (t_0_), bacterial growth was only marginally affected across the tested concentrations. After 2 h of exposure (t_2_), a progressive reduction in colony size and recoverability was observed, particularly at concentrations ≥ 0.612 mg mL^−1^. Following prolonged incubation (t_21_), the antimicrobial activity became markedly more pronounced. *Staphylococcus aureus* was consistently more susceptible than *Bacillus cereus*, with almost complete inhibition observed at the highest extract concentrations. No evident differences in antimicrobial efficacy were detected between the two pomegranate peel extracts, suggesting that both cultivars possess similar antibacterial potential.

Gram-negative bacteria (*P. aeruginosa* and *E. coli*) showed sensitivity only after prolonged exposure and at higher concentrations (>20 mg/mL). The inhibitory concentration observed against Gram-positive bacteria (≥0.612 mg/mL) indicates a moderate antibacterial activity, which is consistent with values reported for hydroalcoholic pomegranate peel extracts in the literature [57,58,59]. Although the antimicrobial activity of purified punicalagin and ellagic acid remains controversial, the efficacy of the crude extract is likely attributable to synergistic interactions among ellagitannins and other phenolic constituents. Conversely, the limited activity against *E. coli* and *P. aeruginosa* is consistent with the intrinsic resistance of Gram-negative bacteria, whose outer lipopolysaccharide membrane and efflux systems reduce the penetration and intracellular accumulation of polyphenolic compounds [61].

### 3.8. Study Limitations and Future Perspectives

Despite the promising findings, some aspects deserve further investigation. The present study evaluated two Italian pomegranate cultivars, highlighting the importance of cultivar selection in determining ellagitannin recovery and biological activity. Future studies including additional cultivars and production batches will help to further validate the proposed approach. The dose-dependent effects observed in *C. elegans*, with beneficial responses at intermediate concentrations and reduced efficacy at higher doses, are consistent with a hormetic mechanism and underline the importance of defining appropriate supplementation levels. Although the antimicrobial activity against *Staphylococcus aureus* and *Bacillus cereus* and the favorable cytocompatibility profile support the potential of GH for dermo-cosmetic applications, additional studies in advanced skin models, formulation systems, and mammalian models will be valuable to further confirm its efficacy, safety, and translational potential.

## 4. Conclusions

This study demonstrates that pomegranate peel, an abundant agro-industrial by-product, can be effectively valorized through a sustainable hydroalcoholic extraction process to obtain ellagitannin-rich extracts with relevant biological activity. Among the tested conditions, the hydroalcoholic extract from the Granato cultivar provided the best compromise between extraction yield, phenolic selectivity, and antioxidant capacity using food-grade solvents and mild processing conditions. Sustainable hydroalcoholic extraction efficiently recovered ellagitannin-rich fractions from pomegranate peels, generating extracts with antioxidant, antibacterial, and pro-longevity activities.

Chemical characterization confirmed punicalagin as the major bioactive component, and the strong in vitro antioxidant activity of GH was reflected in vivo by improvements in lifespan and healthspan markers in *C. elegans*. GH supplementation enhanced neuromuscular performance, preserved intestinal barrier integrity, and improved mitochondrial function during aging. Mechanistic investigations revealed activation of conserved stress-response pathways, with SKN-1 playing a key role in the pro-longevity effects of the extract. GH also induced a transient increase in mitochondrial respiratory activity during early adulthood, consistent with an adaptive hormetic modulation of mitochondrial metabolism. Regarding antibacterial activity, it was concentration- and time-dependent, with high efficacy against the tested Gram-positive bacteria. Taken together, these findings highlight pomegranate peel as a sustainable source of ellagitannin-rich bioactive compounds with potential applications in antioxidant nutraceutical and dermo-cosmetic formulations targeting oxidative stress and healthy aging. Although ellagitannin bioavailability was not directly assessed and further validation in mammalian models is needed, these findings highlight pomegranate peel as a promising source of bioactive compounds for nutritional and cosmetic applications within a circular bioeconomy framework.

## Data Availability

The original contributions presented in this study are included in the article/Supplementary Material. Further inquiries can be directed to the corresponding authors.

## Supplementary Materials

The following supporting information can be downloaded at: https://www.mdpi.com/article/10.3390/antiox15081018/s1, Table S1: Primers for real-time qPCR analysis; Figure S1: Chromatographic profile of cultivar G extracts. Aqueous extracts (GA; blue), ethanol extracts (GE; green), and hydroalcoholic extracts (GH; red) were recorded at 360 nm. The figure shows punicalin (PUN), punicalagin (α PU + β PU), ellagic acid (EA); Figure S2: Chromatographic profile of cultivar R extracts. Aqueous extracts (RA; blue), ethanol extracts (RE; green), and hydroalcoholic extracts (RH; red) were recorded at 360 nm. The figure shows: punicalin (PUN), punicalagin (α PU + β PU), ellagic acid (EA); Table S2: HPLC-DAD data related to different pomegranate peel extracts. Values are expressed as mean ± SD (*n* = 3). Different letters indicate statistically significant differences (*p* < 0.05); Table S3: Antioxidant activity of pomegranate peel extracts determined by DPPH and FRAP assays. Values are expressed as mean ± SD (*n* = 3). Different letters indicate statistically significant differences (*p* < 0.05); Table S4: Lifespan analysis of N2 wild-type *C. elegans* supplemented with different concentrations of the hydroalcoholic pomegranate peel extract GH (50 µg/mL to 20 mg/mL); Figure S3: Assessment of cytosolic ROS levels in GH-treated worms. Cytosolic ROS levels in 1- or 10-day-old adults treated with 1 mg/mL GH, compared to the untreated control group (UT). Statistical analysis was evaluated by two-way ANOVA with the Bonferroni post-test (ns: not significant). Bars represent the mean of three independent experiments; Figure S4: Mitochondrial DNA (mtDNA)/chromosomal DNA (chrDNA) ratio. mtDNA content was assessed by quantifying the relative mtDNA to chrDNA ratio using RT-qPCR. mtDNA copy number was calculated by normalizing mtDNA amplification to chrDNA amplification. Data are presented as mean ± standard deviation from three independent experiments. Statistical analysis was performed using two-way ANOVA followed by Bonferroni’s post hoc test (ns: not significant); Figure S5: Translocation of SKN-1 transcription factor mediated by GH in transgenic *C. elegans* strains. Representative fluorescence microscopy images of the *skn-1*:: GFP worm strain at 1 and 5 days of adulthood, after supplementation with 1 mg/mL GH from embryo hatching. Scale bar: 100 µm; Figure S6: Representative spot dilution assay illustrating the antibacterial activity of GH and RH pomegranate peel extracts against *Bacillus cereus* and *Staphylococcus aureus*. Bacteria were treated with increasing extract concentrations (0.019–10 mg mL^−1^), and cells were spotted onto NB plates (t_0_), after 2 h (t_2_), and after 21 h (t_21_). NT, untreated control. Growth was evaluated after 24 h of incubation at 37 °C.
