# Supplementary material for: Sustainable Ellagitannin-Rich Pomegranate Peel Extracts Activate SKN-1/NRF2-Dependent Antioxidant Responses and Promote Healthy Aging in Caenorhabditis elegans
Source: Antioxidants (Basel). 2026 Aug 15;15(8):1018. doi: 10.3390/antiox15081018 (PMC13509578; doi:10.3390/antiox15081018)
Supplement: Supplementary file 1 [file antioxidants-15-01018-s001.zip › antioxidants-4368629-supplementary.pdf]

Sustainable Ellagitannin-Rich Pomegranate Peel Extracts Activate SKN-1/NRF2-Dependent Antioxidant Responses and Promote Healthy Aging in *Caenorhabditis elegans*

Emily Schifano[1], Francesco Cairone[2,*], Marco Guarnieri[3], Andrea Bonfanti[3], Patrizia Mancini[4], Irene Arpante[2], Arianna Montanari[3], Cristina Mazzoni[3], Daniela Uccelletti[3,*], Stefania Cesa[2]

[1] Department for Well-Being, Health and Environmental Sustainability—BeSSA, Sapienza University of Rome, Via della Fontanelle, 02100 Rieti, Italy; emily.schifano@uniroma1.it

[2] Department of Chemistry and Technologies of Drug, Sapienza University of Rome, Piazzale Aldo Moro 5, 00185 Rome, Italy; irene.arpante@uniroma1.it (I.A.); stefania.cesa@uniroma1.it (S.C.)

[3] Department of Biology and Biotechnologies, Sapienza University of Rome, Piazzale Aldo Moro 5, 00185 Rome, Italy; marco.guarnieri@uniroma1.it (M.G.); andrea.bonfanti@uniroma1.it (A.B.); ari.montanari@uniroma1.it (A.M.); cristina.mazzoni@uniroma1.it (C.M.)

[4] Department of Experimental Medicine, Sapienza University of Rome, Viale Regina Elena 324, 00161 Rome, Italy; patrizia.mancini@uniroma1.it

* Correspondence: francesco.cairone@uniroma1.it (F.C.); daniela.uccelletti@uniroma1.it (D.U.)

Table S1. Primers for real time qPCR analysis.

Gene	Forward primer (5′-3′)	Reverse primer (5′-3′)
act-1	GAGCGTGGTTACTCTTTCA	CAGAGCTTCTCCTTGATGTC
cdc-42	CTGCTGGACAGGAAGATTACG	CTCGGACATTCTCGAATGAAG
cox-4	GCCGACTGGAAGAACTTGTC	GCGGAGATCACCTTCCAGTA
daf-2	ATCGTCGGATTCTACTGTACTCCC	CCGACATCTGACAATATTCATTCTC GT
daf-16	TCAAGACCTCAAAGCCAATCAACT C	ACGAGAAAGAAGGAGTAAGAGGA GG
gst-4	TCAATGTGCCTTACGAGGATTA	CGAATTGTTCTCCATCGACTTG
hif-1	TGTGGAATGCGTGCATTTTA	AAAAGCCATTGCAAAACGTC
hsf-1	ATGACTCCACTGTCCCAAGG	TCTTGCCGATTGCTTTCTCT
nd-1	AGCGTCATTTATTGGGAAGAAGAC	AAGCTTGTGCTAATCCCATAAATGT

pmk-1	AAATGACTCGCCGTGATTTC	CATCGTGATAAGCAGCCAGA
sek-1	CAGAGCCGTTTATTGGGAAA	TGCATCCGGCTTGTACAGT
skn-1	GTTCCCAACATCCAACTACG	TGGAGTCTGACCAGTGGATT
sod-3	AGAACCTTCAAAGGAGCTGATG	CCGCAATAGTGATGTCAGAAAG

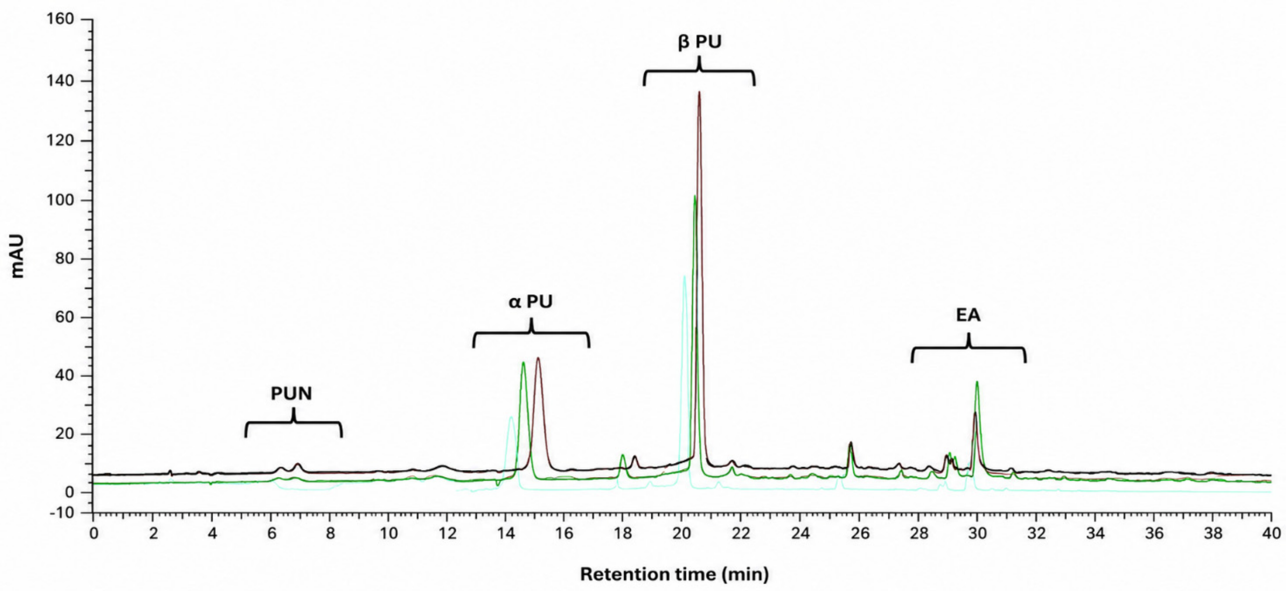

Figure S1. Chromatographic profile of cultivar G extracts. Aqueous extracts (GA; blue), ethanol extracts (GE; green), and hydroalcoholic extracts (GH; red) were recorded at 360 nm. The figure shows punicalin (PUN), punicalagin (α PU + β PU), ellagic acid (EA).

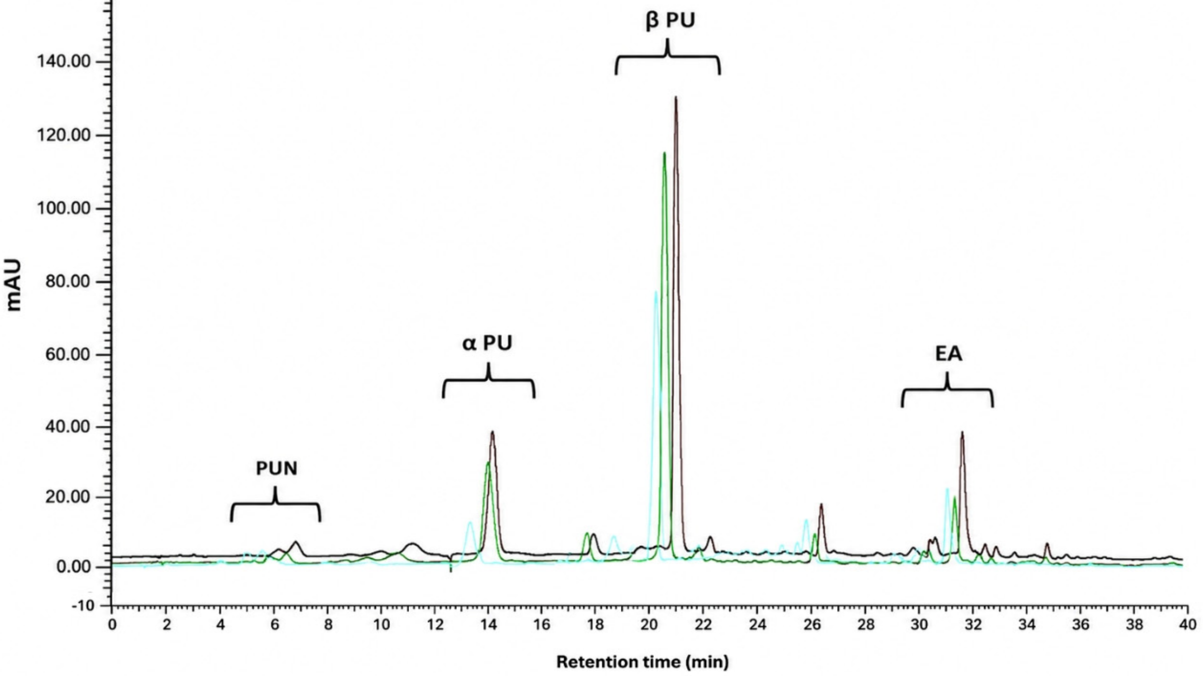

Figure S2. Chromatographic profile of cultivar R. Aqueous extracts (RA; blue), ethanol extracts (RE; green), and hydroalcoholic extracts (RH; red) were recorded at 360 nm. The figure shows : punicalin (PUN), punicalagin (α PU + β PU), ellagic acid (EA).

Table S2. HPLC-DAD data related to different pomegranate peels extracts. Values are expressed as mean ± SD (n = 3). Different letters indicate statistically significant differences ($p < 0.05$).

Samples	PUN mg/g*	PU mg/g*	EA mg/g*	EAD mg/g*	PU/AE ratio
**GA**	nd	$64.71 \pm 0.64^{a**}$	2.61 ± 0.18^{a}	1.15 ± 0.10^{a}	24.70
**RA**	nd	61.19 ± 0.49^{a}	1.15 ± 0.07^{b}	0.4 ± 0.04^{b}	40.79
**GE**	0.84 ± 0.02^{a}	94.75 ± 14.90^{b}	4.62 ± 0.33^{c}	0.97 ± 0.03^{a}	20.50
**RE**	1.03 ± 0.06^{a}	92.56 ± 9.02^{b}	3.25 ± 0.40^{a}	0.89 ± 0.03^{a}	28.48
**GH**	1.62 ± 0.05^{b}	134.94 ± 5.40^{c}	4.14 ± 0.18^{c}	0.88 ± 0.06^{a}	24.98

**RH**	1.21 ± 0.47^a	116.19 ± 8.90^b	5.89 ± 0.82^d	1.87 ± 0.34^c	19.72

*Values are reported in mg/g dry extract; nd = non detectable; PU = punicalin; PUN = punicalagin; EA = ellagic acid; EAD = ellagic acid derivatives. **Different letters indicate statistically significant differences ($p < 0.05$).

Table S3. Antioxidant activity of pomegranate peel extracts determined by DPPH and FRAP assays. Values are expressed as mean ± SD (n = 3). Different letters indicate statistically significant differences ($p < 0.05$).

**Samples**	**DPPH**		**FRAP**	
	**µg/mL***	**mg/g* DE**	**µg/mL§**	**mg/g§ DE**
**GA**	4.60 ± 0.20^a	92.4 0± 4.60^a	1.01 ± 0.03^a	40.32 ± 1.21^a
**RA**	3.20 ± 0.20^a	65.20 ± 4.60^a	1.03 ± 0.03^a	41.41 ± 1.24^a
**GE**	9.60 ± 0.50^b	193.70 ± 9.80^b	0.96 ± 0.02^a	38.77 ± 0.96^a
**RE**	8.20 ± 0.40^b	163.90 ± 8.20^c	1.07 ± 0.06^a	43.11 ± 2.58^a
**GH**	9.30 ± 0.50^b	186.10 ± 5.60^b	1.24 ± 0.12^b	49.77 ± 4.97^b
**RH**	9.40 ± 0.30^b	188.90 ± 5.70^b	1.39 ± 0.08^b	55.41 ± 3.32^b

DE: dry extract; *gallic acid equivalents; §ellagic acid equivalents

Table S4. Lifespan analysis of N2 wild-type *C. elegans* supplemented with different concentrations of the hydroalcoholic pomegranate peel extract GH (50 µg/mL to 20 mg/mL).

GH concentration	Number of individuals	Statistical differences from control	Median lifespan (50% of populace) ± SD	Maximum lifespan
10 µg/mL	240	ns	8 ± 0.05	13 ± 0.18
50 µg/mL	240	ns	9 ± 0.1	14 ± 0.1
100 µg/mL	240	ns	9 ± 0.12	14 ± 0.09
500 µg/mL	240	***	13 ± 0.08	15 ± 0.03
1 mg/mL	240	***	13 ± 0.2	20 ± 0.05
10 mg/mL	240	ns	9 ± 0.1	14± 0.15
20 mg/mL	240	***	4 ± 0.05	11± 0.06

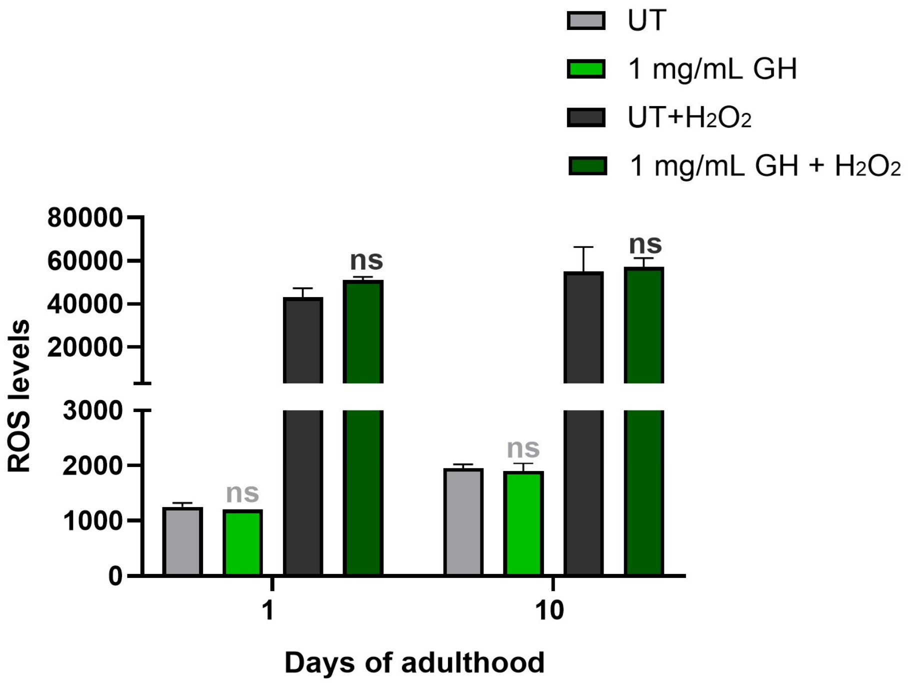

Figure S3. Assessment of cytosolic ROS levels in GH-treated worms. Cytosolic ROS levels in 1 or 10-day-old adults treated with 1 mg/mL GH, compared to the untreated control group (UT). Statistical analysis was evaluated by two-way ANOVA with the Bonferroni post-test (ns: not significant). Bars represent the mean of three independent experiments.

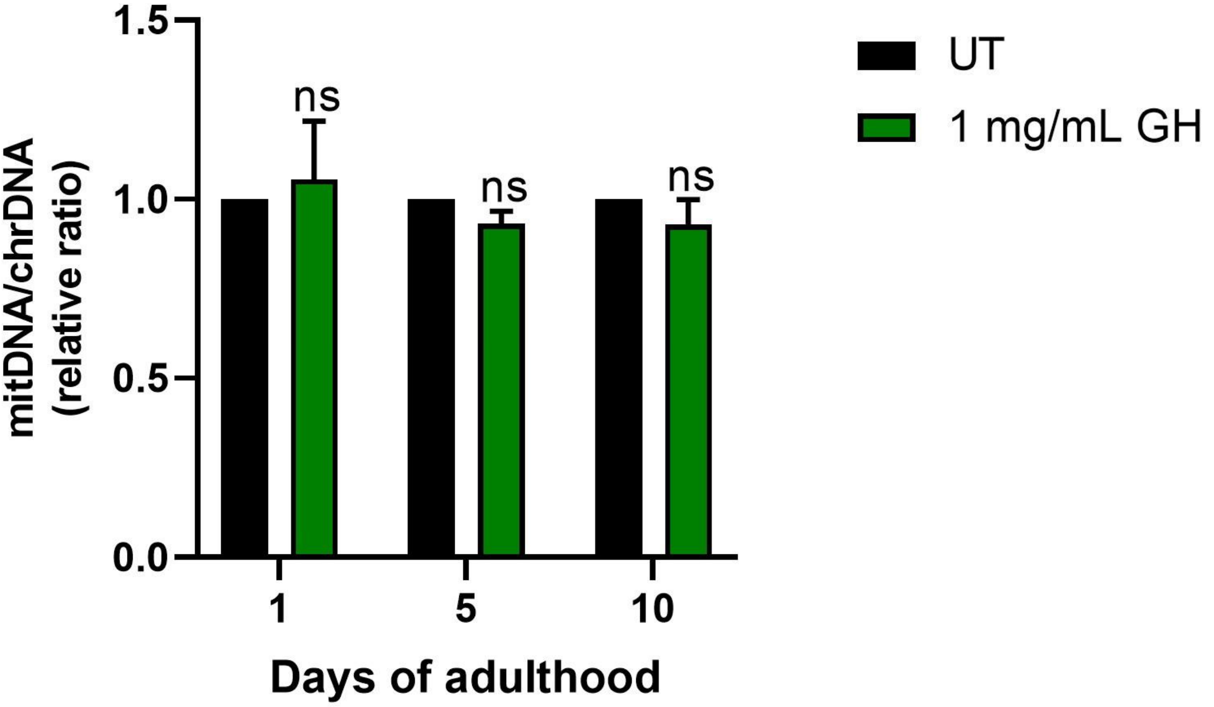

Figure S4. Mitochondrial DNA (mtDNA)/ chromosomal DNA (chrDNA) ratio. mtDNA content was assessed by quantifying the relative mtDNA to chrDNA ratio using RT-qPCR. mtDNA copy number was calculated by normalizing mtDNA amplification to chrDNA amplification. Data are presented as mean ± standard deviation from three independent experiments. Statistical analysis was performed using two-way ANOVA followed by Bonferroni's post hoc test (ns: not significant).

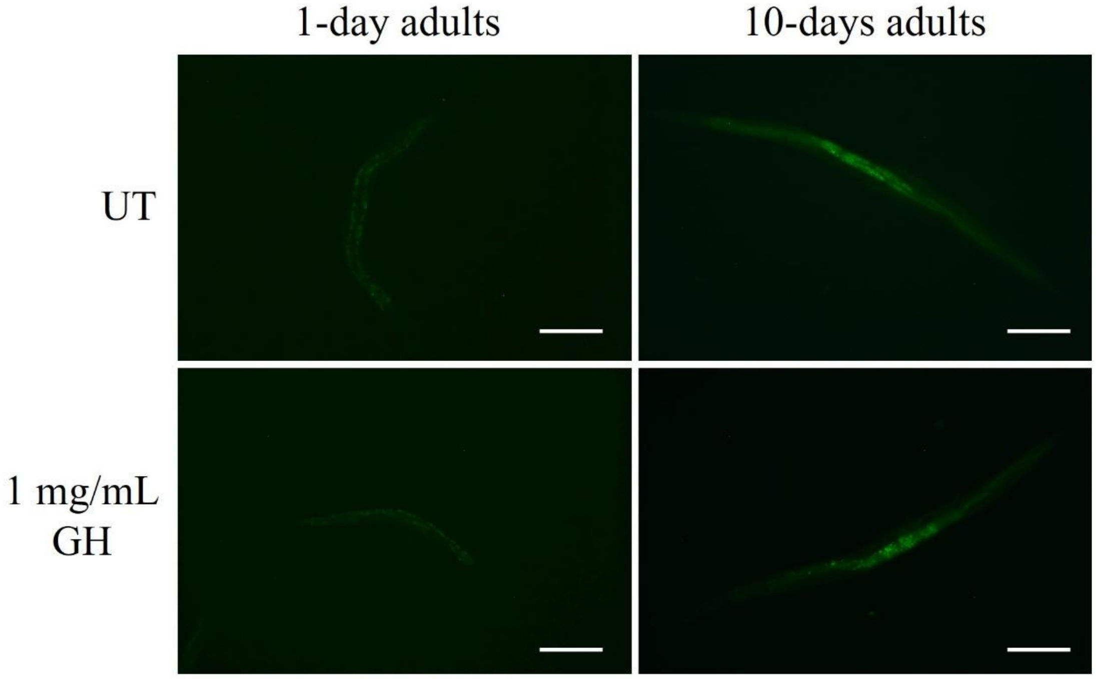

Figure S5. Translocation of SKN-1 transcription factor mediated by GH in transgenic *C. elegans* strains. Representative fluorescence microscopy images of the *skn-1*:: GFP worm strain at 1 and 5 days of adulthood, after supplementation with 1 mg/mL GH from embryos hatching. Scale bar:100 µm.

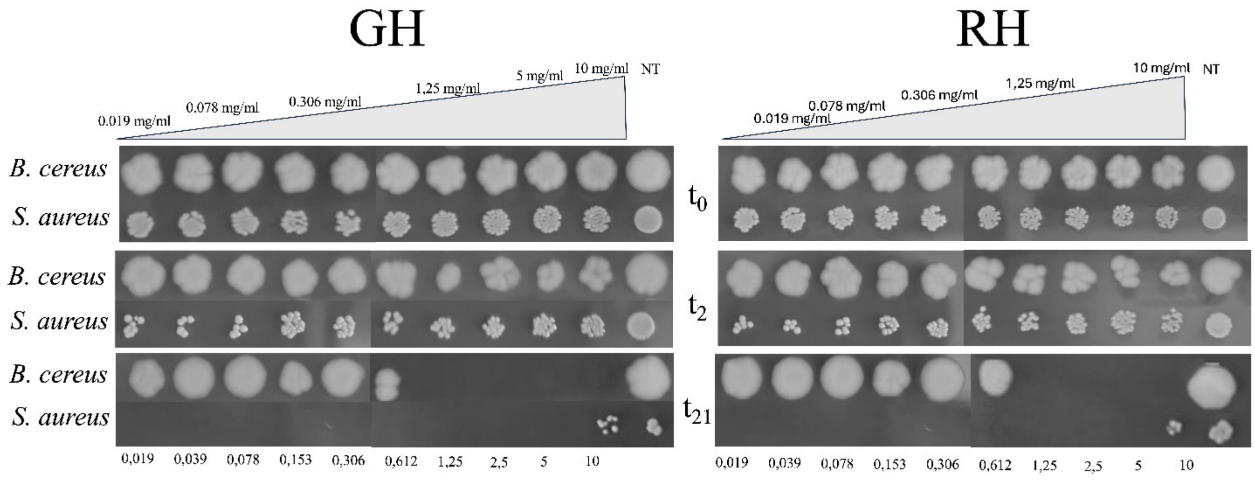

Figure S6. Representative spot dilution assay illustrating the antibacterial activity of GH and RH pomegranate peel extracts against *Bacillus cereus* and *Staphylococcus aureus*. Bacteria were treated with increasing extract concentrations (0.019–10 mg mL^{-1}), cells were spotted onto NB plates (t_0), after 2 h (t_2), and after 21 h (t_{21}). NT, untreated control. Growth was evaluated after 24h of incubation at 37°C
